# A nomogram based on collagen signature for predicting the immunoscore in colorectal cancer

**DOI:** 10.3389/fimmu.2023.1269700

**Published:** 2023-09-14

**Authors:** Wei Jiang, Xian Yu, Xiaoyu Dong, Chenyan Long, Dexin Chen, Jiaxin Cheng, Botao Yan, Shuoyu Xu, Zexi Lin, Gang Chen, Shuangmu Zhuo, Jun Yan

**Affiliations:** ^1^Department of General Surgery, Guangdong Provincial Key Laboratory of Precision Medicine for Gastrointestinal Tumor, Nanfang Hospital, The First School of Clinical Medicine, Southern Medical University, Guangzhou, China; ^2^School of Science, Jimei University, Xiamen, Fujian, China; ^3^Key Laboratory for Biorheological Science and Technology of Ministry of Education (Chongqing University), Chongqing University Cancer Hospital & Chongqing Cancer Institute & Chongqing Cancer Hospital, Chongqing, China; ^4^Division of Colorectal & Anal Surgery, Department of Gastrointestinal Surgery, Guangxi Medical University Cancer Hospital, Nanning, China; ^5^Department of Radiology, Sun Yat-sen University Cancer Center, Guangzhou, China; ^6^Department of Pathology, The Affiliated Cancer Hospital of Fujian Medical University, Fujian Provincial Cancer Hospital, Fuzhou, China; ^7^Precision Medicine Center, Fujian Provincial Cancer Hospital, Fuzhou, China

**Keywords:** immunoscore, colorectal cancer, tumor microenvironment, collagen signature, chemotherapy benefit

## Abstract

**Objectives:**

The Immunoscore can categorize patients into high- and low-risk groups for prognostication in colorectal cancer (CRC). Collagen plays an important role in immunomodulatory functions in the tumor microenvironment (TME). However, the correlation between collagen and the Immunoscore in the TME is unclear. This study aimed to construct a collagen signature to illuminate the relationship between collagen structure and Immunoscore.

**Methods:**

A total of 327 consecutive patients with stage I-III stage CRC were included in a training cohort. The fully quantitative collagen features were extracted at the tumor center and invasive margin of the specimens using multiphoton imaging. LASSO regression was applied to construct the collagen signature. The association of the collagen signature with Immunoscore was assessed. A collagen nomogram was developed by incorporating the collagen signature and clinicopathological predictors after multivariable logistic regression. The performance of the collagen nomogram was evaluated via calibration, discrimination, and clinical usefulness and then tested in an independent validation cohort. The prognostic values of the collagen nomogram were assessed using Cox regression and the Kaplan−Meier method.

**Results:**

The collagen signature was constructed based on 16 collagen features, which included 6 collagen features from the tumor center and 10 collagen features from the invasive margin. Patients with a high collagen signature were more likely to show a low Immunoscore (Lo IS) in both cohorts (*P*<0.001). A collagen nomogram integrating the collagen signature and clinicopathological predictors was developed. The collagen nomogram yielded satisfactory discrimination and calibration, with an AUC of 0.925 (95% CI: 0.895-0.956) in the training cohort and 0.911 (95% CI: 0.872-0.949) in the validation cohort. Decision curve analysis confirmed that the collagen nomogram was clinically useful. Furthermore, the collagen nomogram-predicted subgroup was significantly associated with prognosis. Moreover, patients with a low-probability Lo IS, rather than a high-probability Lo IS, could benefit from chemotherapy in high-risk stage II and stage III CRC patients.

**Conclusions:**

The collagen signature is significantly associated with the Immunoscore in the TME, and the collagen nomogram has the potential to individualize the prediction of the Immunoscore and identify CRC patients who could benefit from adjuvant chemotherapy.

## 1 Introduction

The incidence rate of colorectal cancer (CRC) has gradually increased over the past decades and has become one of the leading causes of cancer burden and cancer deaths worldwide (1). Currently, the tumor-node-metastasis (TNM) staging system is widely utilized in the clinic as the reference standard for prognosis and treatment (2). Nevertheless, there is significant heterogeneity in the clinical outcomes of CRC patients with the same stage who receive a similar treatment regimen. This suggests that the current TNM staging system does not supply adequate prognostic and chemotherapy benefit information (3, 4). Several studies have demonstrated that the tumor microenvironment (TME), including the extracellular matrix (ECM) and immune cells, intensely impacts tumor initiation, proliferation, invasion, and metastasis (5, 6). Among the immune effector cells in the tumor, tumor-infiltrating lymphocytes (TILs) reflect the antitumor immune status of the host and are related to the prognosis and therapeutic response of CRC patients (7, 8). The density of CD3+ and CD8+ T cells at the tumor center (TC) and invasive margin (IM) was quantified and scored, namely, the Immunoscore (9, 10). Recently, several high-quality international studies have validated the prognostic value of the Immunoscore (11–14). Thus, the Immunoscore has been described as a new element for the TNM staging system of CRC and is recommended by the NCCN guidelines (15).

Epithelial-mesenchymal transition (EMT) is known to enhance the migratory and invasive abilities of cancer cells, thereby facilitating tumor formation and metastasis (16). Collagen, as a major component of the extracellular matrix (ECM), is upregulated during the process of EMT under the influence of various transcription factors, such as Twist, Slug, Snail, and Zeb (17–19). Concurrently, the integrins α1β1 and α2β1, which interact with collagen and have been shown to mediate the degradation of epithelial cadherin complexes, are also upregulated (20). Previous research indicated that the interaction between cells and the ECM is regulated through ECM-binding proteins, such as SPARC, which promotes the interaction between collagen and α2β1 (21). SPARC has been demonstrated to induce EMT by regulating SLUG expression and is associated with increased invasiveness (22). Thus, under the influence of various biological signals, the structure of collagen undergoes dynamic changes during the development and progression of tumors (23, 24). Collagen also plays a vital role in the localization, dynamic behavior, and function of TILs in the TME (25, 26). However, the correlation between collagen structure alterations and the Immunoscore remains unclear. Multiphoton imaging, which is a nonlinear optical imaging method, can visualize collagen structure at the supramolecular level and is especially sensitive to collagen structure due to its physical basis (27). This technique has become a powerful tool for investigating the alteration of collagen structure during disease progression (28, 29). Furthermore, our previous studies have established a robust framework that enables automatic high-throughput acquisition of fully quantitative collagen structure features for disease diagnosis and prediction (30–32). Therefore, we hypothesized that we could elucidate the relationship between collagen structure and Immunoscore in the TME of CRC patients using multiphoton imaging and collagen quantification analysis.

Integrating multiple biomarkers into a biomarker panel using a machine learning algorithm can significantly improve the prediction performance compared to individual biomarkers (33, 34). Least absolute shrinkage and selection operator (LASSO) regression is an effective algorithm for analyzing high-throughput data and is widely accepted for model construction (35). Hence, we aimed to construct a fully quantitative collagen biomarker, i.e., a collagen signature, via multiphoton imaging and LASSO regression to comprehensively describe the correlation between collagen structure and the Immunoscore in the TME. Then, we investigated the potential predictive ability of a collagen nomogram that integrated the collagen signature and clinicopathological predictors for individualized prediction of Immunoscore in CRC patients.

## 2 Materials and methods

### 2.1 Patients and tissue specimens

Ethics approval was obtained from the institutional review boards of NanFang Hospital and Fujian Provincial Cancer Hospital (NFEC-2023-221). The requirement for informed consent was waived for this study. The study was conducted following the guidelines of the Declaration of Helsinki and the Transparent reporting of a multivariable prediction model for individual prognosis or diagnosis (TRIPOD) statement criteria.

The flow chart of patient recruitment in this study is shown in **Supplementary Figure 1**. The inclusion criteria were patients who underwent radical surgery with pathologically diagnosed stage I-III CRC, available follow-up data and clinicopathological characteristics, and hematoxylin and eosin (HE) slides with invasive tumor components. The exclusion criteria were patients with unavailable formalin-fixed paraffin-embedded (FFPE) specimens, a history of cancer, or received neoadjuvant treatment. As a result, a total of 327 consecutive patients were included in the training cohort between January 2011 and December 2013 from Nanfang Hospital. An independent validation cohort contained 327 consecutive patients from Fujian Provincial Cancer Hospital between October 2011 and December 2013. Two independent pathologists reassessed all samples based on the 8th edition AJCC staging criteria.

Clinicopathological characteristics included age, sex, primary tumor location, preoperative carcinoembryonic antigen (CEA) level, preoperative carbohydrate antigen 199 (CA199) level, tumor differentiation, tumor size, pT stage, and pN stage. Adjuvant chemotherapy after radical surgery is recommended for patients with high-risk stage II and stage III CRC according to NCCN guidelines.

A standardized follow-up protocol was implemented, including a serum CEA test every 3 months after surgery and every 6 months after 3 years; CT examination from chest to pelvis every 6 months in the first 5 years after surgery; and colonoscopy at 1 year after surgery.

### 2.2 Immunohistochemistry and immunoscore construction

FFPE samples were cut into 4-µm sections and stained with antibodies against CD3 and CD8 (Maixin Biotech. Co., Ltd., Fuzhou, China). Immunohistochemical staining was performed as previously described (36, 37). Whole slide images of stained slices were digitized by Aperio ImageScope (Leica Biosystems, CA, USA) at 20× magnification as.svs format files.

**Figure 1:**
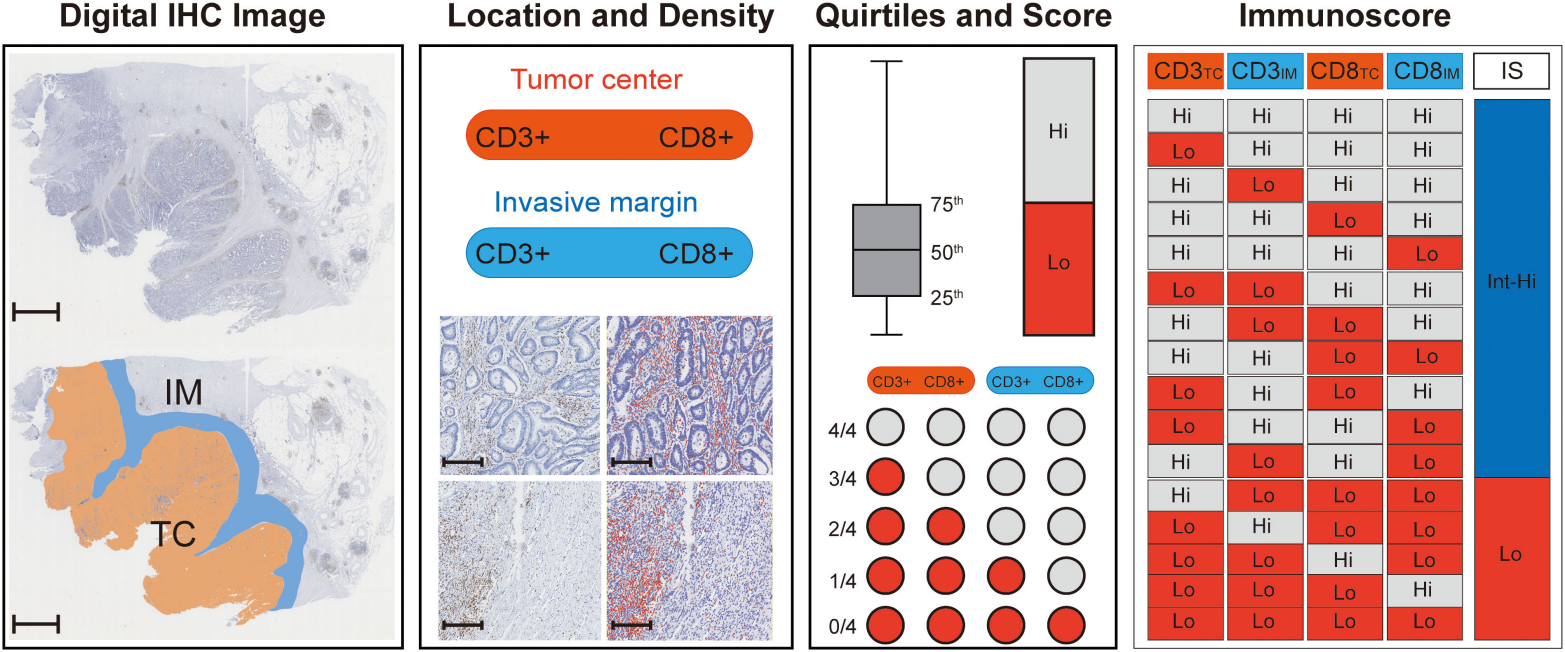
Flowchart for calculating Immunoscore. First, digital IHC images (CD3+ for example) were acquired and opened with Qupath software, and 5 representative images were randomly circled in the TC (orange) and IM (blue) regions (scale: 2,000 μm). Then, the densities of CD3+ (brown) in the CT and IM were counted by Qupath software (red), and the number of positive TILs was calculated per mm^2^, scale: 250 μm. The mean TIL density was used to divide the individual cases into “high” or “low” immune groups, and patients with a mean density ≥ 75th percentile were regarded as a “high” immune group. A high immune group score was set as 1, and a low immune group score was set as 0. The CD3_TC_, CD3_IM_, CD8_TC_, and CD8_IM_ scores were added and converted into an Immunoscore (I0 - I4), where I0-I1 is a low Immunoscore (Lo IS) and 2-4 is an intermediate-high Immunscore (Int-Hi IS). TC, tumor center; IM, invasive margin; IHC, immunohistochemistry; TILs, tumor-infiltrating lymphocytes; IS, Immunoscore; Lo, low; Int-Hi, intermediate-high.

The Immunoscore was assessed in the following steps (**Figure 1**). First, two pathologists who were blinded to the prognostic information selected five representative regions at the TC and five representative regions at the IM. Second, CD3+ and CD8+ stained immune cells were quantified using QuPath software (version 0.2.3). Third, CD3+ and CD8+ density was used to divide the individual cases into “high” or “low” immune groups, and patients with a mean density ≥ 75th percentile were considered a “high” immune group. A high immune group score was set as 1, and a low immune group score was set as 0. The CD3_TC_, CD3_IM_, CD8_TC_, and CD8_IM_ scores were added and converted into an Immunoscore (I0 - I4). Finally, patients were divided into two groups based on their Immunoscore: I0–I1 was classified as low Immunoscore (Lo IS), and I2-I4 was classified as intermediate-high Immunoscore (Int-Hi IS).

### 2.3 Multiphoton imaging and collagen feature extraction

The regions at the TC and IM, which were used to calculate the density of CD3+ and CD8+, were used for multiphoton imaging. Image acquisition for multiphoton imaging was performed with a 200× original magnification objective on another unstained serial section and then compared with the HE image for histologic assessment (27). More information about the multiphoton imaging system can be found in the **Supplementary Methods**.

The framework we constructed for the quantitative extraction of collagen features is shown in the **Supplementary Methods**. In summary, 142 collagen features (**Supplementary Table 1**), including morphological features, histogram-based features, gray level concurrence matrix (GLCM) features, and Gabor wavelet transform features, were achieved automatically via MATLAB 2016b (Mathworks, Natick, MA, USA) (30–32). Finally, a total of 284 collagen features were obtained, including 142 from TC and 142 from IM, for further statistical analyses.

### 2.4 LASSO regression and collagen signature construction

LASSO regression, which is suitable for the regression of high-dimensional data, was used to select the most useful predictive features (33–35). The LASSO regression used an L1 penalty to shrink the coefficients to zero. The penalty parameter λ, also called the tuning constant, controls the number of collagen features to enter the model. In this study, we applied 10-fold cross-validations to select the optimal value of λ via 1-standard error (SE) criteria in the training cohort, and the collage signature was calculated for each patient via a linear combination of selected features that were weighted by their respective coefficients in the training cohort. Then, the collage signature in the validation cohort was calculated by the selected features with their respective coefficients obtained from the training cohort. Details of the LASSO regression are provided in the **Supplementary Methods**.

### 2.5 Development and assessment of the collagen nomogram

The collagen signature and clinicopathologic characteristics were included in univariate analysis to investigate their association with Lo IS, and variables with *P* < 0.10 were included in multivariable analysis. A backward stepwise selection method with Akaike’s information criterion as the stopping rule was used to select the independent predictors of Lo IS (38). To facilitate clinical application, we developed a collagen nomogram according to the independent predictors in the training cohort (39).

The Hosmer−Lemeshow test was applied to estimate the goodness of fit of the model (40). The multicollinearity of the collagen nomogram was evaluated through the variance inflation factor (VIF) (41). The area under the curve (AUC) and the calibration curve were applied to assess the discrimination and calibration of the collagen nomogram. Then, the collagen nomogram was performed in the validation cohort, and its AUC and calibration curve were acquired. More information on the nomogram is shown in the **Supplementary Methods**.

### 2.6 Clinical application value of the collagen nomogram

To assess the clinical application value of the collagen nomogram, a traditional model was developed for comparison with the collagen nomogram. In our study, the traditional model was constructed based on clinicopathological predictors after univariate and multivariable logistic regression in the training cohort. The clinical usefulness of the collagen nomogram was evaluated by decision curve analysis (DCA) and clinical impact curves (CICs) (42). The maximum Youden index value of the ROC curve of the two models was measured to estimate the specificity, sensitivity, accuracy, positive predictive value (PPV), and negative predictive value (NPV). Moreover, the net reclassification improvement (NRI) and integrated discrimination improvement (IDI) were used to show the improvement of the collagen nomogram compared with the traditional model (43, 44). Details of the NRI and IDI are provided in the **Supplementary Methods**.

### 2.7 Statistical analysis

Baseline characteristics were compared between the training and validation cohorts by t test, *U* test, Fisher’s exact test, and χ2 test when applicable. The odds ratio (OR) and 95% confidence interval (CI) of the predictors were calculated using multivariable logistic regression. Survival curves were generated by using the Kaplan–Meier method and compared by log-rank tests. Univariate and multivariable analyses with Cox proportional hazards regression determined the hazard ratio (HR) of predictors for disease-free survival (DFS) and overall survival (OS). All statistical analyses were performed with SPSS version 22.0 software (IBM, Armonk, New York USA) and R version 4.0.3 (http://www.r-project.org/). All *P* values were two-sided, and statistical significance was defined as *P* < 0.05.

## 3 Results

### 3.1 Patient characteristics and immunoscore

The baseline characteristics of the patients in the training and validation cohorts are summarized in **Table 1**. A total of 421 (64.3%) patients were < 65 years old, with 405 (61.9%) men. The clinicopathological characteristics of the two cohorts were similar (**Supplementary Table 2**).

**Table 1:** Clinicopathological characteristics of the patients in the training and validation cohorts.

Characteristic	Training cohort (*n* = 327)		*P*	Validation cohort (*n* = 327)		P
	Lo IS (*n* = 112)	Int-Hi IS (*n* = 215)		Lo IS (*n* = 117)	Int-Hi IS (*n* = 210)	
**Age, years**			0.284			0.376
≥ 65	78 (69.6)	137 (63.7)		70 (59.8)	136 (64.8)	
< 65	34 (30.4)	78 (36.3)		47 (40.2)	74 (35.2)	
**Sex**			0.660			0.310
Male	70 (62.5)	129 (60.0)		64 (54.7)	127 (60.5)	
Female	42 (37.5)	86 (40.0)		53 (45.3)	83 (39.5)	
**Primary tumor location**			0.823			0.698
Left-sided	65 (58.0)	122 (56.7)		70 (59.8)	121 (57.6)	
Right-sided	47 (42.0)	93 (43.3)		47 (40.2)	89 (42.4)	
**Preoperative CEA level**			0.127			0.081
Normal	71 (63.4)	154 (71.6)		72 (61.5)	149 (71.0)	
Elevated	41 (36.6)	61 (28.4)		45 (38.5)	61 (29.0)	
**Preoperative CA19-9 level**			0.225			0.115
Normal	93 (83.0)	189 (87.9)		97 (82.9)	187 (89.0)	
Elevated	19 (17.0)	26 (12.1)		20 (17.1)	23 (11.0)	
**Tumor differentiation**			<0.001			<0.001
Well or moderately	72 (64.3)	179 (83.3)		73 (62.4)	173 (82.4)	
Poorly or undifferentiated	40 (35.7)	36 (16.7)		44 (37.6)	37 (17.6)	
**Tumor size, cm**			0.098			0.059
< 4	46 (41.1)	109 (50.7)		48 (41.0)	109 (51.9)	
≥ 4	66 (58.9)	106 (49.3)		69 (59.0)	101 (48.1)	
**pT stage**			0.013			<0.001
pT1-T3	82 (73.2)	182 (84.3)		78 (66.7)	177 (84.3)	
pT4	30 (26.8)	33 (15.3)		39 (33.3)	33 (15.7)	
**pN stage**			<0.001			0.002
pN0	44 (39.3)	133 (61.9)		49 (41.9)	125 (59.5)	
pN+	68 (60.7)	82 (38.1)		68 (58.1)	85 (40.5)	
**Collagen signature,** **median (IQR)**	3.018 (-0.493, 3.481)	-0.988 (-1.223, -0.800)	<0.001	3.086 (0.867, 3.580)	-1.103 (-1.434, -0.799)	<0.001

Values in parentheses are percentages unless indicated otherwise.

The P value was derived from the univariable association analyses between each of the clinicopathological characteristics and IS.

Lo, low; Int-Hi, intermediate-high; IS, Immunoscore; IQR, interquartile range; CEA, carcinoembryonic antigen; CA199, carbohydrate antigen 199.

The density of CD3+ and CD8+ TILs in the TC and IM is shown in **Supplementary Figure 2**, with a higher density of TILs in the IM than in the TC for both CD3+ and CD8+ cells. The cutoff values of CD3+ and CD8+ cells were 593 and 382 cells/mm^2^ in the TC and 1382 and 714 cells/mm^2^ in the IM, respectively (**Supplementary Table 3**). Finally, the proportions of patients with Lo IS and Int-Hi IS were 34.3% and 65.7% in the training cohort and 35.8% and 64.2% in the validation cohorts, respectively.

**Figure 2:**
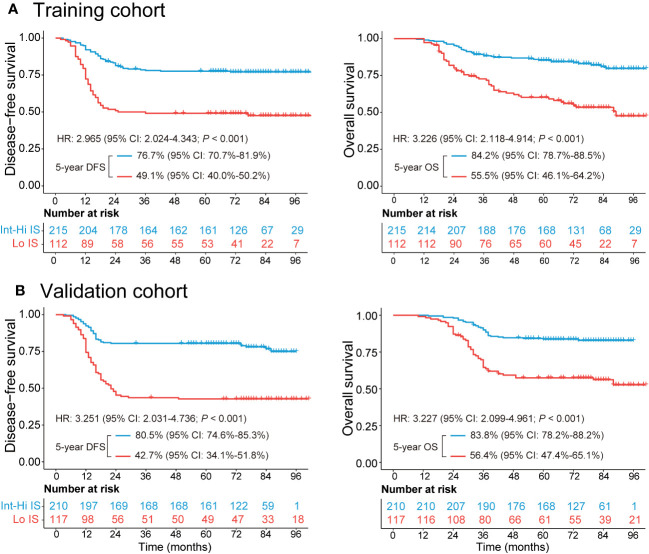
Kaplan−Meier survival analysis of the training and validation cohorts grouped by Immunoscore. **(A)** The 5-year DFS and OS comparison between the Lo and Int-Hi IS groups in the training cohort. **(B)** The 5-year DFS and OS comparison between the Lo and Int-Hi IS groups in the validation cohort. Lo, low; Int-Hi, intermediate-high; IS, Immunoscore; DFS, disease-free survival; OS, overall survival; HR, hazard ratio.

The median follow-up duration [interquartile range (IQR)] was 72 (42–85) months in the training cohort and 71 (40–83) months in the validation cohort. The 5-year DFS and OS rates were 67.6% and 74.6%, respectively, in the training cohort. Similarly, the DFS and OS rates were 67.0% and 74.3%, respectively, in the validation cohort (**Supplementary Figure 3**). Patients with Int-Hi IS from the training cohort had a significantly better 5-year DFS (76.7% vs. 49.1%; *P <* 0.001) and 5-year OS (84.2% vs. 55.5%; *P <* 0.001) than patients with Lo IS (**Figure 2A**). Likewise, patients with Int-Hi and Lo IS had significant differences in 5-year DFS (80.5% vs. 42.7%; *P <* 0.001) and 5-year OS (83.8% vs. 56.4%; *P <* 0.001) in the validation cohort (**Figure 2B**).

### 3.2 Collagen signature construction

**Figure 3:**
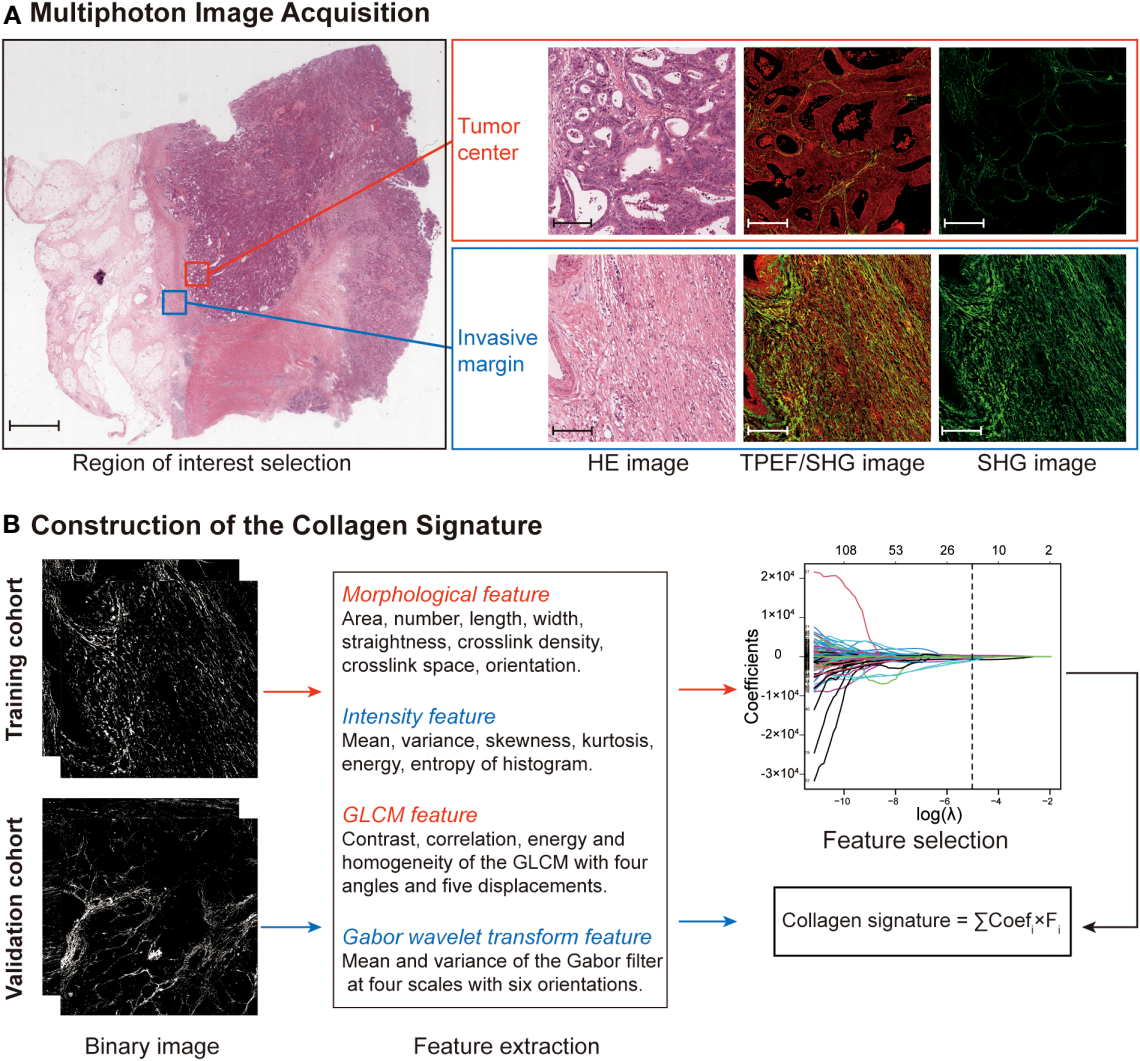
Construction framework of the collagen signature. **(A)** Selection of the region of interest in the TC and IM by comparing HE staining and multiphoton imaging. Ten regions (five at the TC and five at the IM) per sample are used for multiphoton imaging. Scale bars: 2,000 μm and 200 μm, respectively. **(B)** Framework for constructing the collagen signature. SHG images were converted to binary images for collagen feature extraction. The collagen signature was constructed using LASSO regression from 284 collagen features (142 from the TC and 142 from the IM). Then, the relationship between the collagen signature and the Immunoscore was evaluated and validated. HE, hematoxylin and eosin; TPEF, two-photon excitation fluorescence; SHG, second harmonic generation; GLCM, gray-level cooccurrence matrix; LASSO, least absolute shrinkage and selection operator; TC, tumor center; IM, invasive margin.

**Figure 4:**
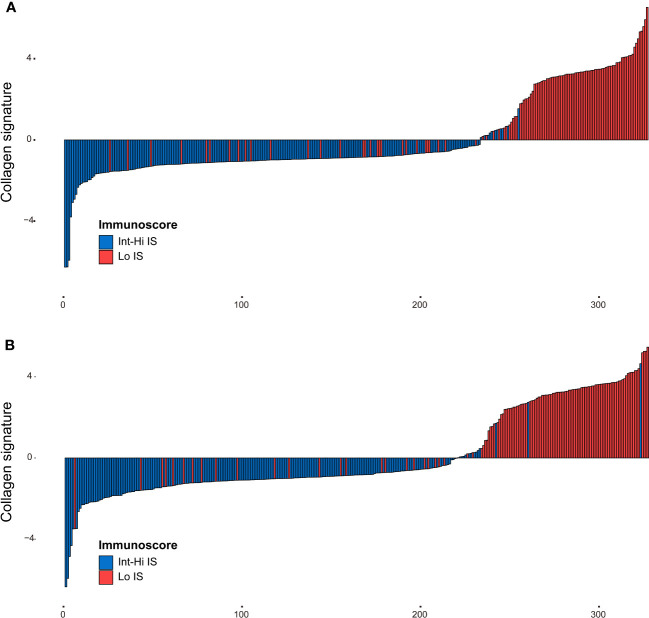
Distribution of the collagen signature in the training and validation cohorts. **(A)** Collagen signature for each patient in the training cohort. **(B)** Collagen signature for each patient in the validation cohort. Red represents the Lo Immunoscore, and blue represents the Int-Hi Immunoscore. Lo, low; Int-Hi, intermediate-high; IS, Immunoscore.

The framework of the collagen signature is presented in **Figure 3**. As a result, a collagen signature was constructed based on sixteen collagen predictors from 284 collagen features by LASSO regression. (**Supplementary Figure 4**). The calculation formula for the collagen signature is proposed in the **Supplementary Results**. The distributions of the 16 collagen predictors and Immunoscore for each patient in the training and validation cohorts are shown in **Supplementary Figure 5**. The patients with a high collagen signature were more likely to show Lo IS in both cohorts (**Figure 4****)**. The collagen signature yielded an AUC of 0.896 (95% CI, 0.854-0.936) in the training cohort and 0.903 (95% CI, 0.863-0.944) in the validation cohort. A significant association between the collagen signature and Lo IS was found when stratified analysis was performed (**Supplementary Tables 4**, **5**).

**Figure 5:**
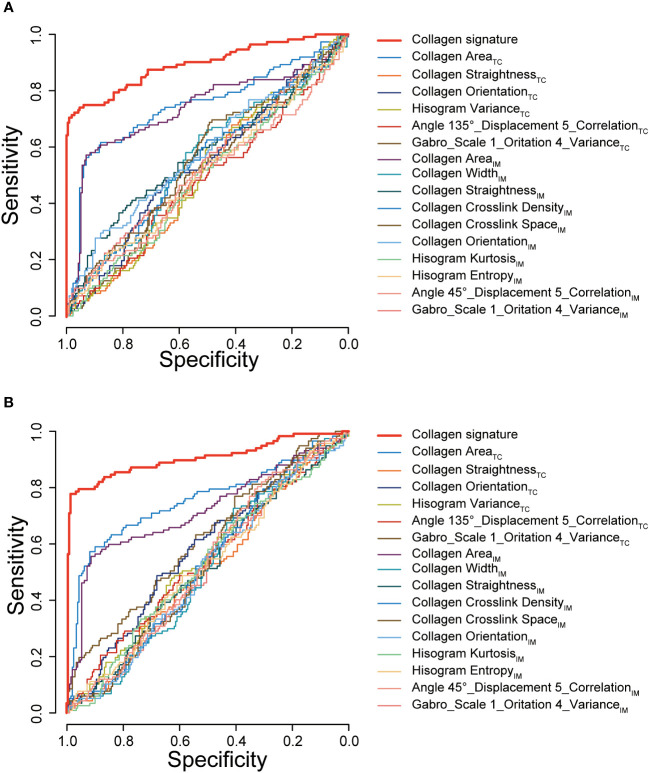
ROC curves of the collagen signature and the single selected collagen features. ROC curves of the collagen signature and the 16 selected collagen features in predicting Immunoscore in the training cohort **(A)** and validation cohort **(B)**. TC, tumor center; IM, invasive margin.

We also assessed the performance of the collagen signature and the single selected collagen feature in predicting Immunoscore. The results indicated that the collagen signature was more powerful than any individual parameter, demonstrating the added predictive value of the collagen signature (**Figure 5**).

### 3.3 Development and validation of the collagen nomogram

**Figure 6:**
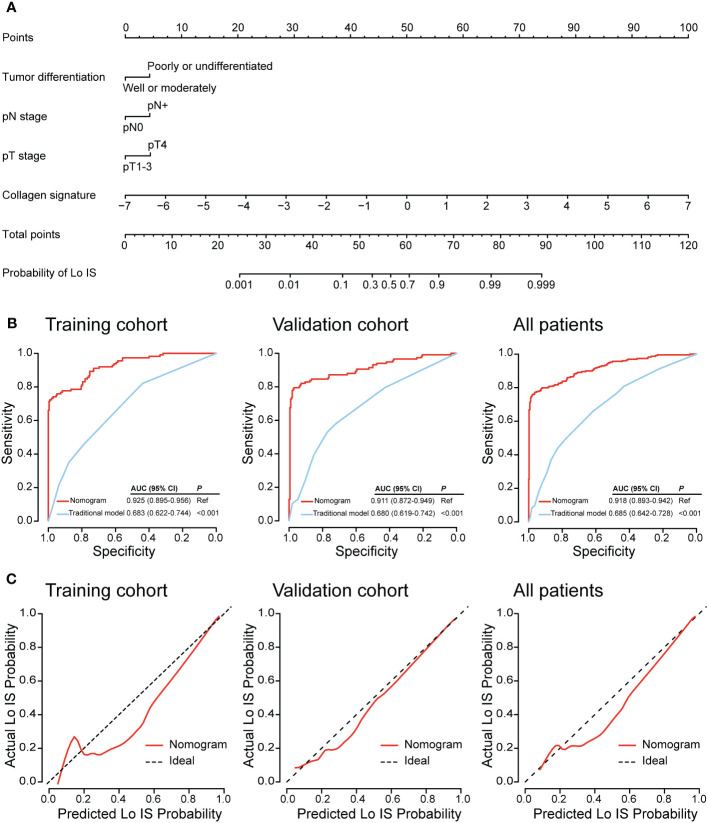
Collagen nomogram construction and performance assessment. **(A)** The collagen nomogram was constructed in the training cohort, incorporating the collagen signature, tumor differentiation, pT stage, and pN stage. **(B)** The ROC curves of the nomogram and the traditional model in the training cohort, the validation cohort, and all patients. **(C)** The calibration curves of the nomogram in the training cohort, the validation cohort, and all patients. In the calibration curve, the y-axis represents the actual Lo IS probability, and the x-axis represents the predicted Lo IS probability. The diagonal black dotted line represents a perfect prediction model. The solid red line is a representation of the nomogram; better prediction is indicated when the solid red line has a closer fit to the diagonal black dotted line. AUC, area under the curve; CI, confidence interval; Lo IS, low Immunoscore.

Univariate and multivariable logistic regression was performed to identify independent predictors of Lo IS. The results showed that the collagen signature (OR: 4.632, 95% CI: 3.068-6.993; *P* < 0.001), tumor differentiation (OR: 2.537, 95% CI: 1.121-5.741; *P* = 0.026), pT stage (OR: 2.602, 95% CI: 1.106-6.121; *P* = 0.028), and pN stage (OR: 2.550, 95% CI: 1.197-5.433; *P* = 0.015) were independent predictors of Lo IS (**Table 2**). Then, the collagen nomogram was developed, integrating the above four predictors (**Figure 6A**). ROC curve analysis indicated that the collagen signature had the most discrimination ability compared with the other predictors (**Supplementary Figure 6**). Alluvial diagrams were employed to intuitively illustrate the relationship between the four predictors and Immunoscore (**Supplementary Figure 7**). The variance inflation factor (VIF) values of each predictor were < 10, indicating that there was no multicollinearity among the four predictors (**Supplementary Table 6**). The Hosmer−Lemeshow test yielded a nonsignificant statistic (*P* = 0.299), demonstrating that there was no departure from a perfect fit.

**Table 2:** Univariate and multivariable analyses of the predictors of Lo IS in the training cohort.

Variables	Univariate analysis		Multivariable analysis	
	OR (95% CI)	*P*	OR (95% CI)	*P*
Age, years				
≥ 65	Ref			
< 65	1.306 (0.801, 2.131)	0.285		
Sex				
Male	Ref			
Female	0.900 (0.563, 1.440)	0.660		
Primary tumor location				
Left-sided	Ref			
Right-sided	0.949 (0.597, 1.506)	0.823		
Preoperative CEA level				
Normal	Ref			
Elevated	1.458 (0.897, 2.369)	0.128		
Preoperative CA19-9 level				
Normal	Ref			
Elevated	1.485 (0.782, 2.821)	0.227		
Tumor differentiation				
Well or moderately	Ref		Ref	
Poorly or undifferentiated	2.762 (1.631, 4.678)	<0.001	2.537 (1.121, 5.741)	0.026
Tumor size, cm				
< 4	Ref			
≥ 4	1.475 (0.930, 2.341)	0.099	NA	NA
pT stage				
pT1-3	Ref		Ref	
pT4	2.018 (1.154, 3.529)	0.014	2.602 (1.106, 6.121)	0.028
pN stage				
pN0	Ref		Ref	
pN+	2.507 (1.569, 4.005)	<0.001	2.550 (1.197, 5.433)	0.015
**Collagen signature**	4.596 (3.075, 6.870)	<0.001	4.632 (3.068, 6.993)	<0.001

Lo IS, low Immunoscore; OR, odds ratio; CI, confidence interval; CEA, carcinoembryonic antigen; CA199, carbohydrate antigen 199; NA, not available; Ref, reference.

In the training cohort, the collagen nomogram yielded satisfactory discrimination with an AUC of 0.925 (95% CI: 0.895-0.956). The calibration curve showed good agreement between the predicted and the actual Lo IS probability (**Figure 6B**). Similar results were observed in the validation cohort (AUC: 0.911, 95% CI: 0.872-0.949) and all patients (AUC: 0.918, 95% CI: 0.893-0.942) (**Figure 6C**).

### 3.4 Clinical application value of the collagen nomogram

**Figure 7:**
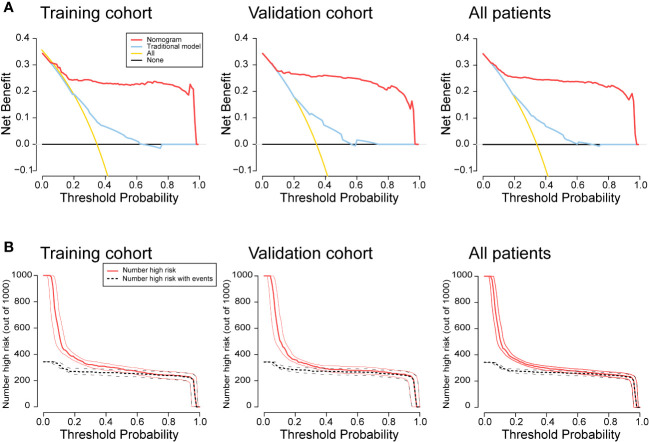
Clinical application value of the nomogram. **(A)** Decision curve analysis for the nomogram. The y-axis represents the net benefit, and the x-axis represents the different threshold probabilities. **(B)** Clinical impact curves for the nomogram. Of 1,000 patients, the red line shows the total number of patients who would be deemed to have a low Immunoscore for each threshold probability. The black line shows how many of those would be true positives (cases). The closer the curves are, the higher the probability that the nomogram would identify low Immunoscore patients from the total estimated number of low Immunoscore patients.

A traditional model was developed based on tumor differentiation, pT stage, and pN stage in the training cohort (**Supplementary Table S7**). The traditional model yielded AUCs of 0.683 (95% CI, 0.622-0.744) in the training cohort, 0.680 (95% CI, 0.619-0.742) in the validation cohort, and 0.685 (95% CI, 0.642-0.728) in all patients. The collagen nomogram exhibited better discrimination ability than the traditional model (training cohort: 0.925 vs. 0.683; validation cohort: 0.911 vs. 0.680; all patients: 0.918 vs. 0.685; all *P* < 0.001) (**Figure 6B**). Moreover, the stratified analysis showed that the collagen nomogram was still superior to the traditional model among the subgroups in the training cohort, the validation cohort, and all patients (**Supplementary Figures S8**–**S10**). DCA revealed that the collagen nomogram could add more benefits than the traditional model (**Figure 7A**). CICs were generated to intuitively recognize the application value of the collagen nomogram to more accurately identify patients with Lo IS (**Figure 7B**).

Furthermore, the collagen nomogram exhibited better sensitivity (97.6% vs. 82.1%), specificity (87.3% vs. 43.7%), accuracy (89.9% vs. 56.6%), PPV (72.3% vs. 43.2%), and NPV (99.1% vs. 82.5%) in the training cohort. Similar results were observed in the validation cohort and all patients (**Table 3**). The corresponding NRI and IDI both showed significantly improved classification accuracy of the collagen nomogram compared with the traditional model in the training cohort, validation cohort and all patients (**Table 4**).

**Table 3:** Predictive power of Lo IS between the nomogram and traditional model.

Model	AUC	Sensitivity(%)	Specificity(%)	Accuracy(%)	PPV(%)	NPV(%)
Training cohort						
Nomogram	0.925 (0.895, 0.956)	97.6 (91.6, 99.6)	87.3 (82.5, 90.9)	89.9 (86.2, 92.7)	72.3 (63.4, 79.8)	99.1 (96.7, 99.8)
Traditional model	0.683 (0.622, 0.744)	82.1 (74.0, 88.1)	43.7 (37.3, 50.4)	56.6 (51.2, 61.9)	43.2 (36.7, 49.9)	82.5 (74.4, 88.3)
Validation cohort						
Nomogram	0.911 (0.872, 0.949)	95.7 (89.6, 98.3)	88.4 (83.7, 91.9)	90.5 (86.9, 93.2)	76.9 (68.5, 83.6)	98.1 (95.2, 99.3)
Traditional model	0.680 (0.619, 0.742)	79.5 (71.3, 85.8)	42.9 (36.4, 49.6)	56.0 (50.5, 61.2)	43.7 (37.2, 50.4)	81.8 (73.6, 87.9)
All patients						
Nomogram	0.918 (0.893, 0.942)	96.6 (92.8, 89.4)	87.8 (84.6, 90.5)	90.2 (87.7, 92.3)	74.7 (86.7, 79.9)	98.6 (97.0, 99.4)
Traditional model	0.685 (0.642, 0.728)	80.8 (75.2, 85.4)	43.3 (38.7, 48.0)	56.4 (52.6, 60.2)	43.4 (38.8, 48.2)	80.7 (75.1, 85.3)

Lo IS, low Immunoscore; AUC, area under the curve; PPV, positive predictive value; NPV, negative predictive value.

**Table 4:** NRI and IDI test for prediction of Lo IS improvements of the nomogram compared with the traditional model.

Models	NRI (95% CI)	*P*	IDI (95% CI)	*P*
Nomogram vs. Traditional model				
Training cohort	0.551 (0.443, 0.660)	<0.001	0.516 (0.445, 0.587)	<0.001
Validation cohort	0.606 (0.496, 0.717)	<0.001	0.532 (0.467, 0.597)	<0.001
All patients	0.564 (0.484, 0.645)	<0.001	0.523 (0.475, 0.571)	<0.001

Lo IS, low Immunoscore; CI, confidence interval; NRI, net reclassification improvement; IDI, integrated discrimination improvement.

### 3.5 Association of the collagen nomogram with prognosis and chemotherapy benefits

**Figure 8:**
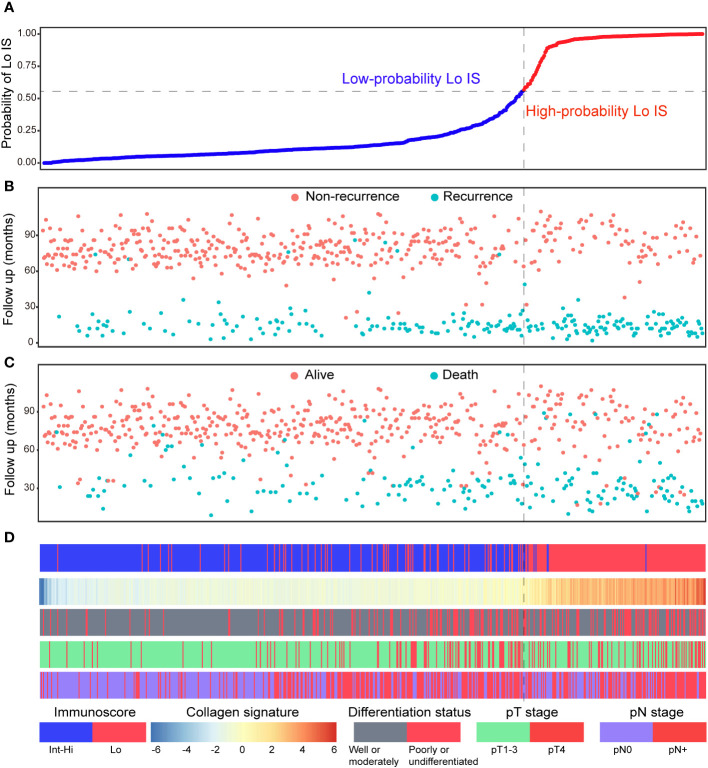
Distribution of the nomogram-predicted subgroups with the corresponding survival status in all patients. **(A)** Nomogram-predicted probability of Lo IS distribution; **(B)** Disease-free survival status of all patients; **(C)** Overall survival status of all patients. **(D)** Distribution of the collagen signature and clinicopathological predictors with the corresponding survival status. Lo IS, low Immunoscore; Int-Hi IS, intermediate-high Immunoscore.

Patients were divided into high- and low-probability Lo IS groups based on the ROC curve of the collagen nomogram. We found that patients with a low-probability Lo IS subgroup showed a better prognosis than patients with a high-probability Lo IS subgroup in the training cohort (**Supplementary Figure S11A**), the validation cohort (**Supplementary Figure S11B**) and all patients (**Supplementary Figure S11C**). This result was also observed in stage I-II (**Supplementary Figure 12**) and III patients (**Supplementary Figure S13**). Cox regression analysis demonstrated that the probability of Lo IS was an independent prognostic factor after adjusting for other variables in the training cohort [DFS: HR 2.475 (95% CI, 1.667-3.675), *P* < 0.001; OS: HR 2.179 (95% CI: 1.409-3.370), *P* < 0.001] (**Supplementary Table 8**), the validation cohort [DFS: HR 2.211 (95% CI, 1.510-3.239), *P* < 0.001; OS: HR 2.111 (95% CI: 1.366-3.262), *P* < 0.001] (**Supplementary Table 9**), and all patients [DFS: HR 2.350 (95% CI, 1.787-3.091), *P* < 0.001; OS: HR 2.119 (95% CI: 1.559-2.881), *P* < 0.001] (**Supplementary Table 10**). The collagen signature and clinicopathological predictors with the corresponding DFS and OS status are presented in **Figure 8**.

**Figure 9:**
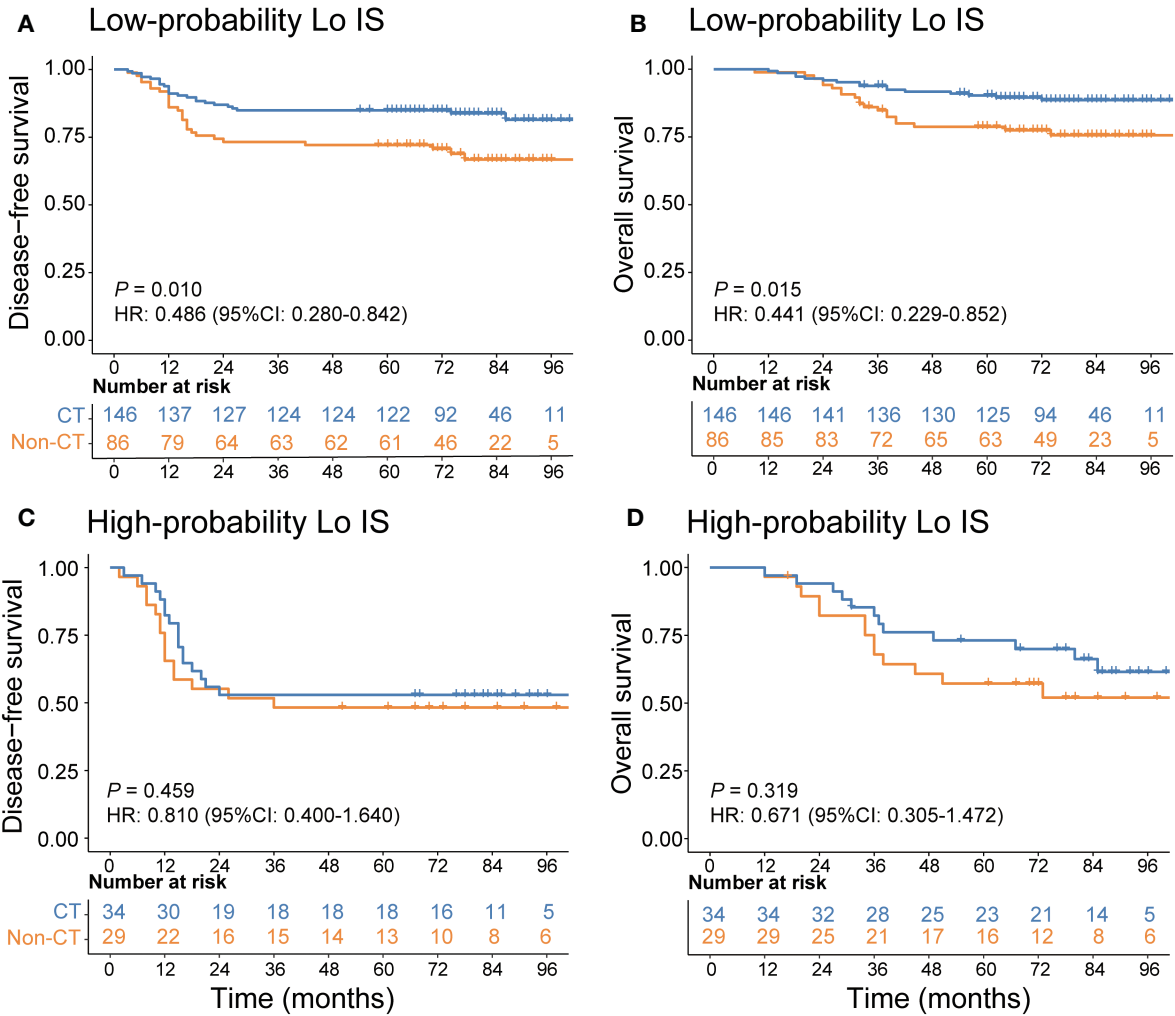
Adjuvant chemotherapy benefits in high-risk stage II CRC patients. **(A)** DFS and **(B)** OS comparison of high-risk stage II CRC according to the receipt of adjuvant chemotherapy in patients with a low-probability Lo IS. **(C)** DFS and **(D)** OS comparison of stage high-risk stage II CRC according to the receipt of adjuvant chemotherapy in patients with a high-probability Lo IS. Lo IS, low Immunoscore; CRC, colorectal cancer; DFS, disease-free survival; OS, overall survival; CT, chemotherapy.

**Figure 10:**
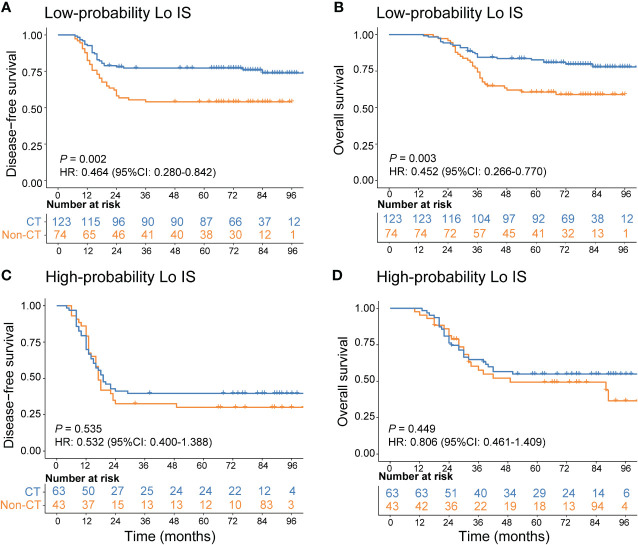
Adjuvant chemotherapy benefits in stage III CRC patients. **(A)** DFS and **(B)** OS comparison of stage III CRC according to the receipt of adjuvant chemotherapy in patients with a low-probability Lo IS. **(C)** DFS and **(D)** OS comparison of stage III CRC according to the receipt of adjuvant chemotherapy in patients with a high-probability Lo IS. Lo IS, low Immunoscore; CRC, colorectal cancer; DFS, disease-free survival; OS, overall survival; CT, chemotherapy.

In addition, we investigated the chemotherapy benefits of high-risk stage II and stage III CRC patients in the high- and low-probability Lo IS subgroups. The results of the survival analysis showed that chemotherapy was associated with high-risk II and stage III CRC patients (**Supplementary Figure S14**). A test for an interaction between the probability of Lo IS and chemotherapy demonstrated that in either high-risk stage II or stage III, the benefit observed in the low-probability Lo IS patients [high-risk stage II (**Figure 9**): DFS, HR: 0.486 (95% CI: 0.280-0.842), *P* = 0.010; OS, HR: 0.441 (95% CI: 0.229-0.852), *P* = 0.015; stage III (**Figure 10**): DFS, HR: 0.464 (95% CI: 0.284-0.758), *P* = 0.002; OS, HR: 0.452 (95% CI: 0.266-0.770), *P* = 0.003; all *P* < 0.05 for interaction; **Table 5**] was superior to that observed in the high-probability Lo IS patients. The results indicated that chemotherapy significantly improved survival outcomes in the low-probability Lo IS group (high-risk stage II: *P* = 0.010 and *P* = 0.015; stage III: *P* = 0.002 and *P* = 0.003, respectively) but had no significant influence in the high-probability Lo IS group (high-risk stage II: *P* = 0.459 and *P* = 0.319; stage III: *P* = 0.535 and *P* = 0.449, respectively).

**Table 5:** Adjuvant chemotherapy interaction with the probability of Lo IS for survival in patients with high-risk stage II and stage III disease.

Probability of Lo IS	Chemotherapy		Disease-free survival		*P*_interaction_	Overall survival		*P*_interaction_
	No CT	CT	HR (95% CI)	*P*		HR (95% CI)	*P*	
High-risk stage II (n = 295)								
Low	86 (37.1)	146 (62.9)	0.486 (0.280, 0.842)	0.010	0.001	0.441 (0.229, 0.852)	0.015	<0.001
High	29 (46.0)	34 (54.0)	0.810 (0.400, 1.640)	0.459		0.671 (0.305, 1.472)	0.319		
Stage III (n = 303)								
Low	74 (37.6)	123 (62.4)	0.464 (0.284, 0.758)	0.002	<0.001	0.452 (0.266, 0.770)	0.003	<0.001
High	43 (40.6)	63 (59.4)	0.859 (0.532, 1.388)	0.535		0.806 (0.461, 1.409)	0.449		

Lo IS, low Immunoscore; CT, chemotherapy; DFS, disease-free survival; OS, overall survival; HR, hazard ratio; CI, confidence interval.

## 4 Discussion

In the current era of precision medicine, Immunoscore is a standard assay that quantifies the density of TILs, and its prognostic value has been internationally validated. In this study, we found a significant association between the collagen signature and the Immunoscore in the TME, and the collagen nomogram combining the collagen signature, tumor differentiation, pT stage, and pN stage could predict the Immunoscore with satisfactory performance. Moreover, the collagen nomogram was able to classify chemotherapy benefits in high-risk stage II and stage III CRC patients, indicating its potential as a tool to predict prognosis and facilitate treatment decision-making.

During tumor development, collagen in the extracellular matrix (ECM) undergoes notable remodeling, which affects the biological behavior of tumor cells, including infiltration, proliferation, and metastasis (18, 19). Importantly, collagen has also been found to influence various types of tumor-infiltrating immune cells (25, 26). In 3D culture assays, T-cell migration was significantly slower in high-density collagen gels than in low-density collagen gels (45). Increased collagen density also results in increased matrix stiffness, which can further affect T-cell migration (46, 47). In addition, high collagen density can influence immunological synapse formation between T cells and antigen-presenting cells (48), leading to reduced T-cell activity (49, 50). Collagen density has also been found to intensely affect the activity of T cells after the initial activation stage (51). These findings suggest that collagen has important immunomodulatory functions, which lays a foundation for quantitatively analyzing the relationship between collagen structure and the Immunoscore in the TME.

Collagen is a noncentrosymmetric structure, and multiphoton imaging can provide detailed information about the structure and organization of collagen fibers in tissue (52, 53). In this study, we acquired high-resolution multiphoton images from the TC and IM of the tumor sample. We then extracted quantitative high-throughput collagen features from the images using a robust framework, which could objectively quantify the collagen structural information contained in the TME. LASSO regression, an effective algorithm with variable selection and complexity regularization, was used to shrink and choose the most predictive collagen predictors from the high-throughput features to construct the collagen signature. Variable selection means selectively choosing variables in the model to achieve more satisfactory performance parameters, rather than including all variables in the model, while complexity regularization is retained through the penalty parameter λ to avoid overfitting (35, 54, 55). Using this approach, the collagen signature, based on 6 collagen features from TC and 10 collagen features from IM, was developed and was significantly related to the Immunoscore. Our findings revealed that patients with a high collagen signature exhibited a low T-cell density microenvironment, resulting in Lo IS in CRC patients with poor prognosis, consistent with previous reports (10, 12, 13). Thus, the collagen signature could comprehensively and quantitatively determine the correlation between collagen structure and Immunoscore in the TME. Then, we constructed a collagen nomogram that included the collagen signature, tumor differentiation, pT stage, and pN stage. The collagen nomogram has better discrimination and clinical application value for estimating the Immunoscore than the traditional model. To the best of our knowledge, this is the first study to assess the association between the collagen structure and the Immunoscore in the TME and build an effective prediction model based on the fully quantitative collagen signature using multiphoton imaging.

From a clinical practice standpoint, the clinical translation of the collagen nomogram is feasible. First, the clinicopathological predictors required for the nomogram are routinely supplied in the postoperative pathological report. Second, unlike immunohistochemistry, which requires staining agents and is time consuming, multiphoton imaging can quickly image unstained sections in a label-free manner, and collagen feature extraction can be automatically completed using MATLAB software. Third, our study revealed a correlation between collagen structure and Immunoscore, indicating that future treatment might regulate collagen in the TME to potentially tune the antitumor immune status. Taken together, we believe that the collagen nomogram is both time efficient for pathologists and cost contained for patients while also providing a potential therapeutic target for improving the prognosis of CRC patients.

According to the NCCN guidelines, adjuvant chemotherapy is recommended for high-risk stage II and stage III CRC patients. However, not all patients can benefit from chemotherapy. Previous clinical trials have shown that patients with Lo IS could not benefit from chemotherapy, while patients with Hi-Int IS could improve their prognosis from chemotherapy; therefore, the Immunoscore is useful for the selection of individualized chemotherapy (12, 13). Because the collagen nomogram demonstrated satisfactory performance in predicting Lo IS, we further evaluated whether the collagen nomogram can identify patients who could benefit from chemotherapy. Patients were divided into high- and low-probability Lo IS groups according to the collagen nomogram. The results showed that patients with a low-probability Lo IS could benefit from chemotherapy, while patients with a high-probability Lo IS could not. This finding suggests that the collagen nomogram could be a potential tool to assist in individualizing chemotherapy selection in high-risk stage II and stage III CRC patients when Immunoscore evaluation is not feasible.

Artificial intelligence (AI) technologies, especially deep learning, have advanced rapidly in medical care, providing powerful methods for constructing accurate prediction models (56, 57). AI has demonstrated comparable performance to pathologists in distinguishing between benign and malignant colorectal diseases (58). Although this approach cannot entirely supplant the role of pathologists, AI can be harnessed as an assistive tool to improve diagnostic efficiency, reduce workload, and improve medical image readability, ultimately reducing the rates of misdiagnosis and missed diagnoses (59). Furthermore, a multistain deep learning model based on AI could also be used to determine the AImmunoscore (AIS) in CRC patients and predict the response to neoadjuvant therapy in rectal cancer patients (60). The potential of AI to revolutionize the clinical landscape of CRC is substantial. However, it is important to recognize that AI is still in its early stages of clinical application in CRC. Several challenges that must be addressed include the validation and generalizability of the predictive models, interpretation of the model, and the safe management and use of data. We believe that in the future, AI technologies will assume a considerably more prominent role in the context of screening, diagnosis, surgical treatment, and prognosis prediction.

Our study has some limitations. First, this was a retrospective study. Second, all specimens were obtained from a single medical center in China. Hence, multicenter, international, prospective clinical trials will be necessary to validate the robustness of the collagen nomogram. Third, the probability of Lo IS based on the collagen nomogram was associated with survival; however, additional survival parameters were not added to our nomogram for model accuracy estimation.

In conclusion, this study proposed that the collagen signature was significantly associated with the Immunoscore in the TME and that the collagen nomogram is useful for the individualized prediction of the Immunoscore in CRC patients. Moreover, the collagen nomogram could be a potential tool to assist in individualizing chemotherapy selection in high-risk stage II and stage III CRC patients.

## Data availability statement

The original contributions presented in the study are included in the article/**Supplementary Material**. Further inquiries can be directed to the corresponding authors.

## Ethics statement

The studies involving humans were approved by ethics approval was obtained from the institutional review boards of NanFang Hospital and Fujian Provincial Cancer Hospital. The studies were conducted in accordance with the local legislation and institutional requirements. The ethics committee/institutional review board waived the requirement of written informed consent for participation from the participants or the participants’ legal guardians/next of kin because this retrospective study met the following criteria: 1) the study involves no more than minimal risk to subjects, and 2) the waiver or alteration will not adversely affect the rights and welfare of the subjects.

## Author contributions

WJ: Conceptualization, Data curation, Formal Analysis, Methodology, Software, Writing – original draft. XD: Conceptualization, Data curation, Methodology, Software, Writing – original draft. XY: Conceptualization, Data curation, Formal Analysis, Resources, Software, Writing – original draft. CL: Data curation, Methodology, Software, Writing – original draft. DC: Data curation, Formal Analysis, Methodology, Software, Writing – original draft. JC: Data curation, Investigation, Software, Writing – review & editing. BY: Data curation, Investigation, Methodology, Software, Writing – original draft. SX: Formal Analysis, Investigation, Methodology, Software, Writing – review & editing. ZL: Formal Analysis, Methodology, Software, Visualization, Writing – review & editing. GC: Conceptualization, Methodology, Supervision, Validation, Visualization, Writing – review & editing. SZ: Conceptualization, Methodology, Software, Supervision, Validation, Visualization, Writing – review & editing. JY: Conceptualization, Formal Analysis, Funding acquisition, Investigation, Methodology, Project administration, Supervision, Visualization, Writing – review & editing.

## References

[1] Sung H Ferlay J Siegel R Laversanne M Soerjomataram I Jemal A. Global cancer statistics 2020: GLOBOCAN estimates of incidence and mortality worldwide for 36 cancers in 185 countries. CA Cancer J Clin (2021) 71(3):209–49. doi: 10.3322/caac.21660 33538338

[2] Delattre JF Selcen Oguz Erdogan A Cohen R Shi Q Emile JF Taieb J. A comprehensive overview of tumour deposits in colorectal cancer: Towards a next TNM classification. Cancer Treat Rev (2022) 103:102325. doi: 10.1016/j.ctrv.2021.102325 34954486

[3] National Cancer Institute’s SEER database. Available at: http://seercancergov/ (Accessed 26 August 2020).

[4] Dienstmann R Mason MJ Sinicrope FA Phipps AI Tejpar S Nesbakken A. Prediction of overall survival in stage II and III colon cancer beyond TNM system: a retrospective, pooled biomarker study. Ann Oncol (2017) 28(5):1023–31. doi: 10.1093/annonc/mdx052 PMC5406760 28453697

[5] Kirchhammer N Trefny MP Auf der Maur P Laubli H Zippelius A. Combination cancer immunotherapies: Emerging treatment strategies adapted to the tumor microenvironment. Sci Transl Med (2022) 14(670):eabo3605. doi: 10.1126/scitranslmed.abo3605 36350989

[6] Xiang X Niu YR Wang ZH Ye LL Peng WB Zhou Q. Cancer-associated fibroblasts: Vital suppressors of the immune response in the tumor microenvironment. Cytokine Growth Factor Rev (2022) 67:35–48. doi: 10.1016/j.cytogfr.2022.07.006 35918210

[7] Aristin Revilla S Kranenburg O Coffer PJ. Colorectal cancer-infiltrating regulatory T cells: functional heterogeneity, metabolic adaptation, and therapeutic targeting. Front Immunol (2022) 13:903564. doi: 10.3389/fimmu.2022.903564 35874729 PMC9304750

[8] Joyce JA Fearon DT. T cell exclusion, immune privilege, and the tumor microenvironment. Science (2015) 348(6230):74–80. doi: 10.1126/science.aaa6204 25838376

[9] Angell HK Bruni D Barrett JC Herbst R Galon J. The immunoscore: colon cancer and beyond. Clin Cancer Res (2020) 26(2):332–9. doi: 10.1158/1078-0432.CCR-18-1851 31413009

[10] Bruni D Angell HK Galon J. The immune contexture and Immunoscore in cancer prognosis and therapeutic efficacy. Nat Rev Cancer (2020) 20(11):662–80. doi: 10.1038/s41568-020-0285-7 32753728

[11] Malka D Lievre A Andre T Taieb J Ducreux M Bibeau F. Immune scores in colorectal cancer: Where are we? Eur J Cancer (2020) 140:105–18. doi: 10.1016/j.ejca.2020.08.024 33075623

[12] Mlecnik B Bifulco C Bindea G Marliot F Lugli A Lee JJ. Multicenter international society for immunotherapy of cancer study of the consensus immunoscore for the prediction of survival and response to chemotherapy in stage III colon cancer. J Clin Oncol (2020) 38(31):3638–51. doi: 10.1200/JCO.19.03205 PMC7605397 32897827

[13] Pages F Mlecnik B Marliot F Bindea G Ou FS Bifulco C. International validation of the consensus immunoscore for the classification of colon cancer: a prognostic and accuracy study. Lancet (2018) 391(10135):2128–39. doi: 10.1016/S0140-6736(18)30789-X 29754777

[14] El Sissy C Kirilovsky A Van den Eynde M Musina AM Anitei MG Romero A. A diagnostic biopsy-adapted immunoscore predicts response to neoadjuvant treatment and selects patients with rectal cancer eligible for a watch-and-wait strategy. Clin Cancer Res (2020) 26(19):5198–207. doi: 10.1158/1078-0432.CCR-20-0337 32669377

[15] NCCN. Clinical practice guidelines in oncology-colon cancer (2022 version 3) . Available at: http://www.nccn.org.

[16] Lamouille S Xu J Derynck R. Molecular mechanisms of epithelial-mesenchymal transition. Nat Rev Mol Cell Biol (2014) 15(3):178–96. doi: 10.1038/nrm3758 PMC4240281 24556840

[17] Ang HL Mohan CD Shanmugam MK Leong HC Makvandi P Rangappa KS. Mechanism of epithelial-mesenchymal transition in cancer and its regulation by natural compounds. Med Res Rev (2023) 43(4):1141–200. doi: 10.1002/med.21948 36929669

[18] Levental KR Yu H Kass L Lakins JN Egeblad M Erler JT. Matrix crosslinking forces tumor progression by enhancing integrin signaling. Cell (2009) 139(5):891–906. doi: 10.1016/j.cell.2009.10.027 19931152 PMC2788004

[19] Mouw JK Ou G Weaver VM. Extracellular matrix assembly: a multiscale deconstruction. Nat Rev Mol Cell Biol (2014) 15(12):771–85. doi: 10.1038/nrm3902 PMC4682873 25370693

[20] Zhang Y Cheng K Xu B Shi J Qiang J Shi S. Epigenetic input dictates the threshold of targeting of the integrin-dependent pathway in non-small cell lung cancer. Front Cell Dev Biol (2020) 8:652. doi: 10.3389/fcell.2020.00652 32793596 PMC7387701

[21] Girotti MR Fernandez M Lopez JA Camafeita E Fernandez EA Albar JP. SPARC promotes cathepsin B-mediated melanoma invasiveness through a collagen I/alpha2beta1 integrin axis. J Invest Dermatol (2011) 131(12):2438–47. doi: 10.1038/jid.2011.239 21850018

[22] Lopez-Moncada F Torres MJ Lavanderos B Cerda O Castellon EA Contreras HR. SPARC Induces E-Cadherin Repression and Enhances Cell Migration through Integrin alphavbeta3 and the Transcription Factor ZEB1 in Prostate Cancer Cells. Int J Mol Sci (2022) 23(11):5874. doi: 10.3390/ijms23115874 35682554 PMC9180154

[23] Han W Chen S Yuan W Fan Q Tian J Wang X. Oriented collagen fibers direct tumor cell intravasation. Proc Natl Acad Sci USA (2016) 113(40):11208–13. doi: 10.1073/pnas.1610347113 PMC5056065 27663743

[24] Martins Cavaco AC Damaso S Casimiro S Costa L. Collagen biology making inroads into prognosis and treatment of cancer progression and metastasis. Cancer Metastasis Rev (2020) 39(3):603–23. doi: 10.1007/s10555-020-09888-5 32447477

[25] Romer AMA Thorseth ML Madsen DH. Immune modulatory properties of collagen in cancer. Front Immunol (2021) 12:791453. doi: 10.3389/fimmu.2021.791453 34956223 PMC8692250

[26] Salmon H Franciszkiewicz K Damotte D Dieu-Nosjean MC Validire P Trautmann A. Matrix architecture defines the preferential localization and migration of T cells into the stroma of human lung tumors. J Clin Invest (2012) 122(3):899–910. doi: 10.1172/JCI45817 22293174 PMC3287213

[27] Yan J Zheng X Liu Z Liu W Lin D Chen D. Multiphoton imaging provides a superior optical biopsy to that of confocal laser endomicroscopy imaging for colorectal lesions. Endoscopy (2019) 51(2):174–8. doi: 10.1055/a-0641-5091 29996151

[28] Skala MC Riching KM Gendron-Fitzpatrick A Eickhoff J Eliceiri KW White JG. *In vivo* multiphoton microscopy of NADH and FAD redox states, fluorescence lifetimes, and cellular morphology in precancerous epithelia. Proc Natl Acad Sci USA (2007) 104(49):19494–9. doi: 10.1073/pnas.0708425104 PMC2148317 18042710

[29] Zoumi A Yeh A Tromberg BJ. Imaging cells and extracellular matrix in *vivo* by using second-harmonic generation and two-photon excited fluorescence. Proc Natl Acad Sci USA (2002) 99(17):11014–9. doi: 10.1073/pnas.172368799 PMC123202 12177437

[30] Chen D Chen G Jiang W Fu M Liu W Sui J. Association of the collagen signature in the tumor microenvironment with lymph node metastasis in early gastric cancer. JAMA Surg (2019) 154(3):e185249. doi: 10.1001/jamasurg.2018.5249 30698615 PMC6439641

[31] Xu S Wang Y Tai DCS Wang S Cheng CL Peng Q. qFibrosis: a fully-quantitative innovative method incorporating histological features to facilitate accurate fibrosis scoring in animal model and chronic hepatitis B patients. J Hepatol (2014) 61(2):260–9. doi: 10.1016/j.jhep.2014.02.015 PMC4278959 24583249

[32] Chen W Dong S Liu X Wang G Xu S Lei S. Association of the collagen signature in the tumor microenvironment with recurrence and survival of patients with T4N0M0 colon cancer. Dis Colon Rectum (2021) 64(5):563–75. doi: 10.1097/DCR.0000000000001907 33538520

[33] Jiang Y Zhang Q Hu Y Li T Yu J Zhao L. ImmunoScore signature: A prognostic and predictive tool in gastric cancer. Ann Surg (2018) 267(3):504–13. doi: 10.1097/SLA.0000000000002116 28002059

[34] Huang Y Liang C He L Tian J Liang C Chen X. Development and validation of a radiomics nomogram for preoperative prediction of lymph node metastasis in colorectal cancer. J Clin Oncol (2016) 34(18):2157–64. doi: 10.1200/jco.2015.65.9128 27138577

[35] Jian H Ma S Zhang CH. Adaptive LASSO for sparse high-dimensional regression. Stat Sin (2008) 18(4):1603–18. doi: 10.1007/s11135-007-9120-4

[36] Kuang DM Wu Y Chen N Cheng J Zhuang SM Zheng L. Tumor-derived hyaluronan induces formation of immunosuppressive macrophages through transient early activation of monocytes. Blood (2007) 110(2):587–95. doi: 10.1182/blood-2007-01-068031 17395778

[37] Xu J Ding T He Q Yu XJ Wu WC Jia WH. An in *situ* molecular signature to predict early recurrence in hepatitis B virus-related hepatocellular carcinoma. J Hepatol (2012) 57(2):313–21. doi: 10.1016/j.jhep.2012.03.027 22521360

[38] Zhang Z. Variable selection with stepwise and best subset approaches. Ann Transl Med (2016) 4(7):136. doi: 10.21037/atm.2016.03.35 27162786 PMC4842399

[39] Balachandran V Gonen M Smith J DeMatteo R. Nomograms in oncology: more than meets the eye. Lancet Oncol (2015) 16(4):e173–80. doi: 10.1016/s1470-2045(14)71116-7 PMC4465353 25846097

[40] Rozeboom PD Henderson WG Dyas AR Bronsert MR Colborn KL Lambert-Kerzner A. Development and validation of a multivariable prediction model for postoperative intensive care unit stay in a broad surgical population. JAMA Surg (2022) 157(4):344–52. doi: 10.1001/jamasurg.2021.7580 PMC8851361 35171216

[41] Cheng J Sun J Yao K Xu M Cao Y. A variable selection method based on mutual information and variance inflation factor. Spectrochim Acta A Mol Biomol Spectrosc (2022) 268:120652. doi: 10.1016/j.saa.2021.120652 34896682

[42] Fitzgerald M Saville B Lewis R. Decision curve analysis. JAMA (2015) 313(4):409–10. doi: 10.1001/jama.2015.37 25626037

[43] Bragg F Trichia E Aguilar-Ramirez D Besevic J Lewington S Emberson J. Predictive value of circulating NMR metabolic biomarkers for type 2 diabetes risk in the UK Biobank study. BMC Med (2022) 20(1):159. doi: 10.1186/s12916-022-02354-9 35501852 PMC9063288

[44] Jiang Y Jin C Yu H Wu J Chen C Yuan Q. Development and validation of a deep learning CT signature to predict survival and chemotherapy benefit in gastric cancer: a multicenter, retrospective study. Ann Surg (2020) 274(6):e1153–61. doi: 10.1097/sla.0000000000003778 31913871

[45] Wolf K Te Lindert M Krause M Alexander S Te Riet J Willis AL. Physical limits of cell migration: control by ECM space and nuclear deformation and tuning by proteolysis and traction force. J Cell Biol (2013) 201(7):1069–84. doi: 10.1083/jcb.201210152 PMC3691458 23798731

[46] Nicolas-Boluda A Vaquero J Vimeux L Guilbert T Barrin S Kantari-Mimoun C. Tumor stiffening reversion through collagen crosslinking inhibition improves T cell migration and anti-PD-1 treatment. Elife (2021) 10:e58688. doi: 10.7554/eLife.58688 34106045 PMC8203293

[47] Tabdanov ED Rodriguez-Merced NJ Cartagena-Rivera AX Puram VV Callaway MK Ensminger EA. Engineering T cells to enhance 3D migration through structurally and mechanically complex tumor microenvironments. Nat Commun (2021) 12(1):2815. doi: 10.1038/s41467-021-22985-5 33990566 PMC8121808

[48] Dustin ML Choudhuri K. Signaling and polarized communication across the T cell immunological synapse. Annu Rev Cell Dev Biol (2016) 32:303–25. doi: 10.1146/annurev-cellbio-100814-125330 27501450

[49] Dustin ML de Fougerolles AR. Reprogramming T cells: the role of extracellular matrix in coordination of T cell activation and migration. Curr Opin Immunol (2001) 13(3):286–90. doi: 10.1016/s0952-7915(00)00217-x 11406359

[50] Gunzer M Schafer A Borgmann S Grabbe S Zanker KS Brocker EB. Antigen presentation in extracellular matrix: interactions of T cells with dendritic cells are dynamic, short lived, and sequential. Immunity (2000) 13(3):323–32. doi: 10.1016/s1074-7613(00)00032-7 11021530

[51] Kuczek DE Larsen AMH Thorseth ML Carretta M Kalvisa A Siersbaek MS. Collagen density regulates the activity of tumor-infiltrating T cells. J Immunother Cancer (2019) 7(1):68. doi: 10.1186/s40425-019-0556-6 30867051 PMC6417085

[52] Campagnola PJ Loew LM. Second-harmonic imaging microscopy for visualizing biomolecular arrays in cells, tissues and organisms. Nat Biotechnol (2003) 21(11):1356–60. doi: 10.1038/nbt894 14595363

[53] Chen X Nadiarynkh O Plotnikov S Campagnola P. Second harmonic generation microscopy for quantitative analysis of collagen fibrillar structure. Nat Protoc (2012) 7(4):654–69. doi: 10.1038/nprot.2012.009 PMC4337962 22402635

[54] Meier L Geer SVD Bhlmann P Zrich ETH. The group Lasso for logistic regression. J R Stat Soc B (2008) 70(1):53–71. doi: 10.2307/20203811

[55] TibshIrani R. The lasso method for variable selection in the Cox model. Stat Med (1997) 16(4):385–95. doi: 10.1002/(SICI)1097-0258(19970228)16:43.0.CO;2-3 9044528

[56] Qiu H Ding S Liu J Wang L Wang X. Applications of artificial intelligence in screening, diagnosis, treatment, and prognosis of colorectal cancer. Curr Oncol (2022) 29(3):1773–95. doi: 10.3390/curroncol29030146 PMC8947571 35323346

[57] Bilal M Nimir M Snead D Taylor GS Rajpoot N. Role of AI and digital pathology for colorectal immuno-oncology. Br J Cancer (2023) 128(1):3–11. doi: 10.1038/s41416-022-01986-1 36183010 PMC9814831

[58] Sharma A Kumar R Yadav G Garg P. Artificial intelligence in intestinal polyp and colorectal cancer prediction. Cancer Lett (2023) 565:216238. doi: 10.1016/j.canlet.2023.216238 37211068

[59] Chlorogiannis DD Verras G-I Tzelepi V Chlorogiannis A Apostolos A Kotis K. Tissue classification and diagnosis of colorectal cancer histopathology images using deep learning algorithms. Is the time ripe for clinical practice implementation? Gastroenterol Review/Przegląd Gastroenterologiczny (2023). doi: 10.5114/pg.2023.130337 PMC10985751 38572457

[60] Foersch S Glasner C Woerl AC Eckstein M Wagner DC Schulz S. Multistain deep learning for prediction of prognosis and therapy response in colorectal cancer. Nat Med (2023) 29(2):430–9. doi: 10.1038/s41591-022-02134-1 36624314

