# Supplementary material for: A nomogram based on collagen signature for predicting the immunoscore in colorectal cancer
Source: Front Immunol. 2023 Sep 14;14:1269700. doi: 10.3389/fimmu.2023.1269700 (PMC10538535; doi:10.3389/fimmu.2023.1269700)
Supplement: Supplementary file 1 [file DataSheet_1.docx]

Supplementary Material

# Supplementary Methods

## Multiphoton imaging system.

The multiphoton microscopic imaging system used in this work has been described previously (1). In brief, an upright microscope (LSM 880, Zeiss, Germany) equipped with a mode-locked femtosecond Ti: sapphire laser (Chameleon Ultra, Coherent) was used to obtain high-resolution images. In this study, 810 nm linearly polarized light was selected for nonlinear optical imaging, and a Plan-Apochromat ×20 objective (NA = 0.8, Zeiss, Germany) was used for focusing the excitation beam into the samples. A 32-channel GaAsP photomultiplier tube array detector was used for collecting second harmonic generation (SHG) signals (green color) in the wavelength range of 387 to 409 nm and for the collection of two-photon excitation fluorescence (TPEF) signals (red color) in the wavelength range from 430 to 708 nm. To obtain a large-scale image, a fine focusing stage is used to translate the samples, and each large-scale image is stitched together from a series of x-y scan images. Each x-y scan image contains 512 × 512 pixels with a data depth of 12 bits. Then, multiphoton images were compared to the H&E images for histological evaluation.

## A framework of collagen feature extraction.

A total of 142 collagen features were extracted, including 8 morphological features and 134 texture features (**Supplementary Table 1**).

***Morphological features***

Eight morphological features were extracted, namely, the collagen area, fiber number, length, width, straightness, crosslink density, crosslink space, and orientation. The SHG image was first segmented into collagen pixels and background pixels using the Gaussian mixture model method (2). The binary collagen mask image was then processed using a fiber network extraction algorithm (3) to trace each collagen fiber in the image and to identify cross-link points, which are defined as connecting points between two or more fibers. Moreover, we quantified an orientation index to reflect collagen alignment based on Fourier transform spectra (4).

***Intensity features***

For intensity features, a histogram-based approach was used. The mean, variation, skewness, kurtosis, energy, and entropy were calculated from the histogram of the SHG pixel intensity distribution.

***Texture features***

We also included 80 gray-level cooccurrence matrix (GLCM)-based texture features and 48 Gabor wavelet transform features in the analysis [5, 6.(5, 6) The contrast, correlation, energy, and homogeneity were calculated from the GLCM with five different displacements of pixels at 1, 2, 3, 4, and 5 and four different directions at 0, 45, 90, and 135 degrees. To calculate the Gabor wavelet transform features, we convolved the SHG image with Gabor filters at five different scales and six different orientations, and the mean and variance of the magnitude of the convolution over the image at each setting were calculated.

## Construction of the collagen signature using LASSO regression.

Least absolute shrinkage and selection operator (LASSO) is a commonly used high-dimensional predictive regression method whose basic idea is to use the L1 penalty to accurately shrink certain regression coefficients to zero and further obtain an interpretable model (7, 8). Its function expression is as follows:

$$\sum_{i=1}^{N} \left[ y_{i}-g\left( \sum_{j=1}^{m} \beta_{j}x_{ij}+\beta_{0} \right) \right]^{2}+\lambda\sum_{j=1}^{m} \left| \beta_{j} \right|$$

where *y_i_* (*y* = 0 or 1) is the outcome of patient *i*, *N* is the number of patients, *g* is the sigmoid function, *m* is the number of selection features used in the model, *β_j_* is the model parameter, *x_ij_* is the *j*_th_ (*j* = 1, 2, …, *d*) collagen feature of the *i_th_* patient, and λ is the penalty parameter. The sigmoid function *g* is defined as follows:

$$g\left( z \right)= \frac{1}{1+e^{-z}}$$

The penalty parameter λ (also called the adjustment parameter) controls the amount of contraction. The larger the λ value is, the fewer the number of greater predictor contributions to the model are ​​selected. LASSO has been extended and widely used in logistic regression models for high-dimensional data analysis. In addition, LASSO can also be used to optimize the selection of markers in high-dimensional data to avoid overfitting by controlling the complexity of the model. Hence, we adopted the penalized logistic regression model with the LASSO penalty to simultaneously achieve shrinkage and variable selection. Ten-time cross-validations were used to determine the optimal values of λ via 1-SE (standard error) criteria. As a result, a value λ = 0.006618952 with log (λ) = -5.017818 was selected. The optimal tuning parameter resulted in 16 nonzero coefficients, which were used to construct the collagen signature (**Supplementary Figure 4**).

## Visualization of the results of logistic regression via nomogram.

A nomogram is a visualization of the results of logistic regression (9, 10). According to the size of the regression coefficients of predictors, a score is given to each level of each independent variable. For each patient, a total score can be calculated. Then, the probability of the occurrence of the outcome for each patient can be calculated by the conversion function between the score and the probability of the occurrence of the outcome.

## Decision curve analysis of the collagen nomogram.

Decision curve analysis (DCA) is a simple and easy-to-understand mathematical model that judges the availability and benefits of the prediction model by calculating the net benefits under different threshold probabilities (11-13).

In this study, DCA was used to evaluate the prediction model for low Immunoscore. By analyzing the actual results and model predictions, the net benefit was calculated using the following formula:

$$\text{Net }\text{benefit=}\frac{\text{True positive count}}{\text{n}}\text{-}\frac{\text{False positive count}}{\text{n}}\left( \frac{\text{P}_{\text{t}}}{\text{1-}\text{P}_{\text{t}}} \right)$$

In this formula, *P_t_* is the threshold probability of low Immunoscore, and *n* is the total number of patients.

## Integrated discrimination improvement and net reclassification improvement.

Integrated discrimination improvement (IDI) and net reclassification improvement (NRI) are used to compare the diagnostic capabilities of two prediction models and whether one model improves the diagnostic accuracy of the other. Therefore, IDI and NRI are receiving more attention from experts (14, 15). The calculation formulas of IDI and NRI are as follows:

IDI =（IS_new_ – IS_old_）-（IP_new_ – IP_old_）

NRI =（IS_new_- IS_old_）+（IP_new_ - IP_old_）

IS: Integral sensitivity over all possible cutoff values

IP: 1-specificity under different classification threshold settings

The larger the IDI or NRI is, the better the prediction ability of the new model. If IDI or NRI is > 0, it is a positive improvement, indicating that the prediction ability of the new model is improved compared with the old model. If IDI or NRI is < 0, it is considered that the prediction ability of the new model is decreased compared with the old model. If IDI or NRI is = 0, it is considered that the new model is not improved.

## R packages used in this study

The AUC was calculated using the ‘pROC’ package. The calibration curve was plotted using the ‘rms’ package. DCA was conducted using the ‘rmda’ package. LASSO regression was performed using the ‘glmnet’ package. The survival analysis was performed using the ‘survival’ package. The alluvial diagram was plotted using the ‘ggalluvial’ package. The NRI and IDI were calculated using the ‘PredictABEL’ package. The heatmap was plotted using the ‘pheatmap’ package.

# Supplementary Results

Collagen signature = 275.7552 × Collagen Area_TC_

-1007.036 × Collagen Straightness_TC_

+ 53.61806 × Collagen Orientation_TC_

-16.9779 × Hisogram Variance_TC_

- 38.51576 × Angle 135°_Displacement 5_Correlation_TC_

- 356.515 × Gabro_Scale 1_Oritation 4_Variance_TC_

+ 337.0298 × Collagen Area_IM_

-10.86567 × Collagen Width_IM_

- 551.0817 × Collagen Straightness_IM_

-983.6416 × Collagen Crosslink Density_IM_

+ 0.9610342 × Collagen Crosslink Space_IM_

+ 30.22321 × Collagen Orientation_IM_

+0.0007541291 × Hisogram Kurtosis_IM_

-31.27090 × Hisogram Entropy_IM_

+7.473479 × Angle 45°_Displacement 5_Correlation_IM_

- 731.0256 × Gabro_Scale 1_Oritation 4_Variance_IM_

+ 1441.20331

# Supplementary Figures and Tables

## Supplementary Figures


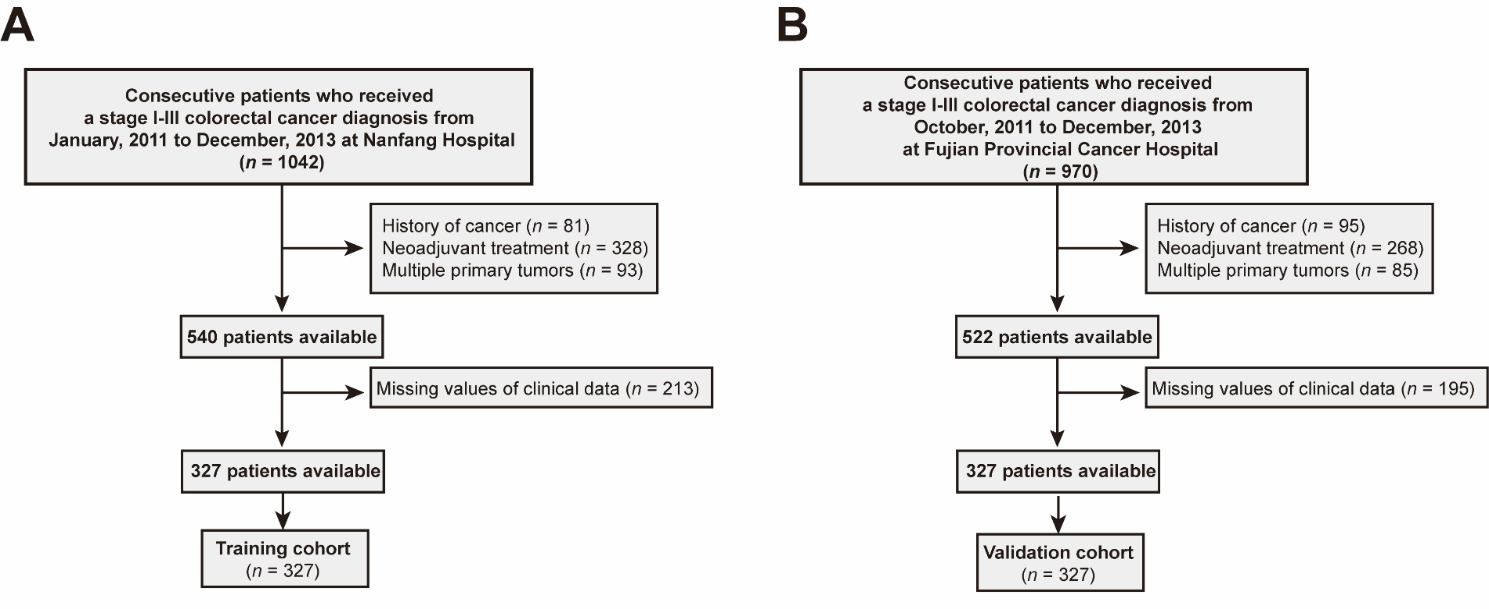


### **Supplementary Figure 1.** Flow chart of the patient recruitment in the training (A) and validation (B) cohorts.


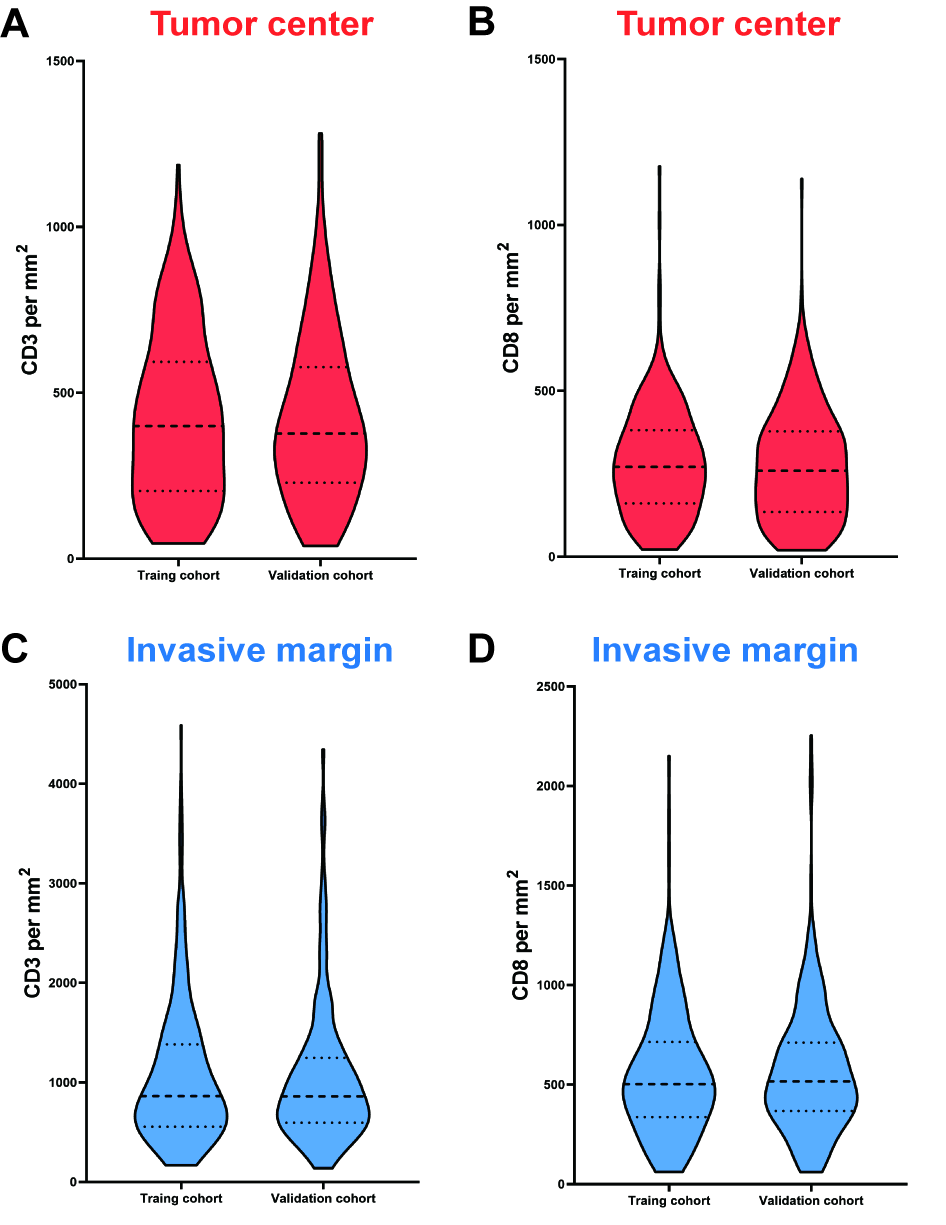


### **Supplementary Figure 2. Distribution of the number of CD3 + and CD8 + TILs in the tumor center and invasive margin.**

*Abbreviations*: TILs, tumor-infiltrating lymphocytes.


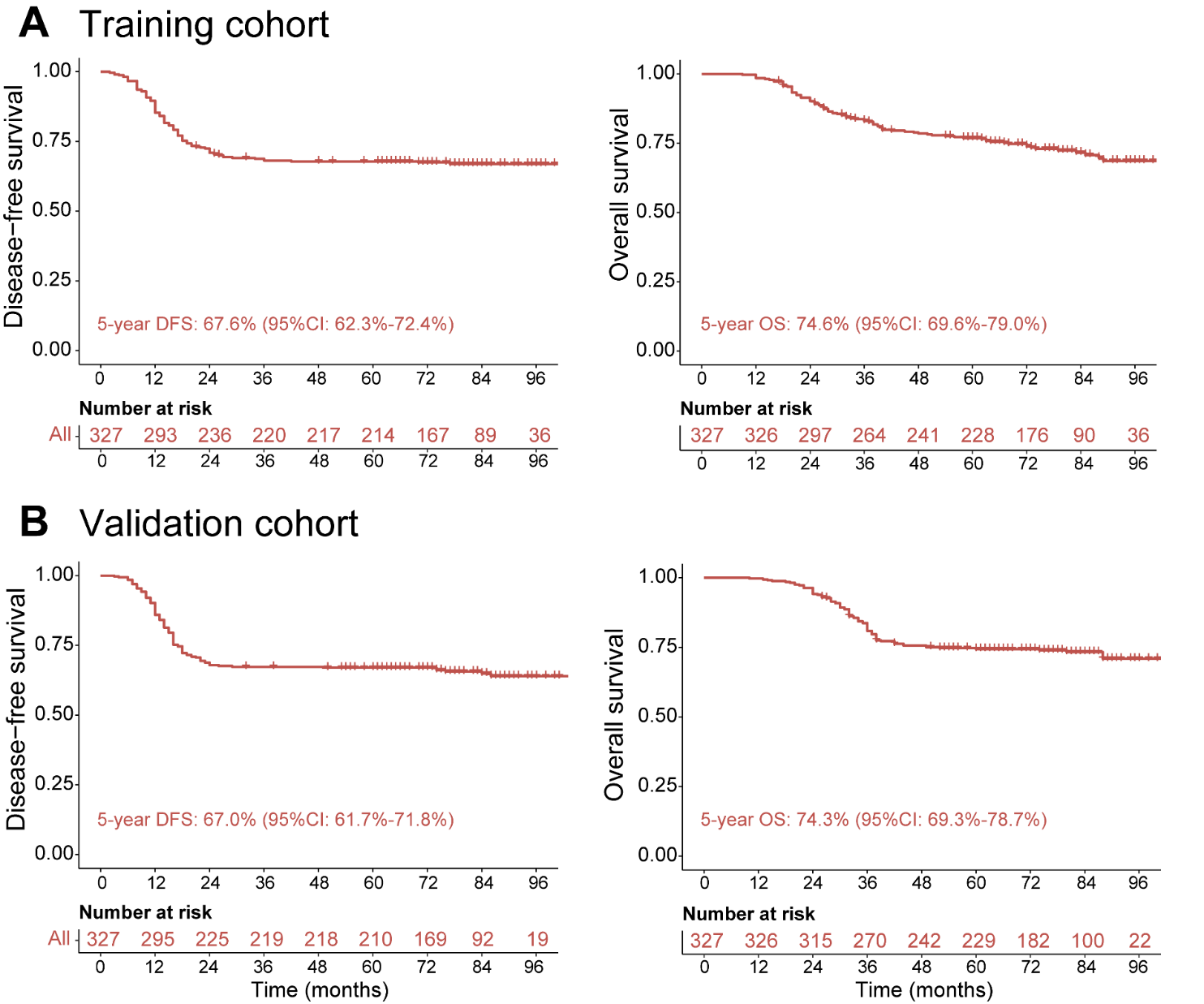


### **Supplementary Figure 3. Kaplan‒Meier survival analysis of the training and validation cohorts.**

**(A)** The DFS curve and OS curve in the training cohort. **(B)** The DFS curve and OS curve in the validation cohort. *Abbreviations*: DFS, disease-free survival; OS, overall survival.


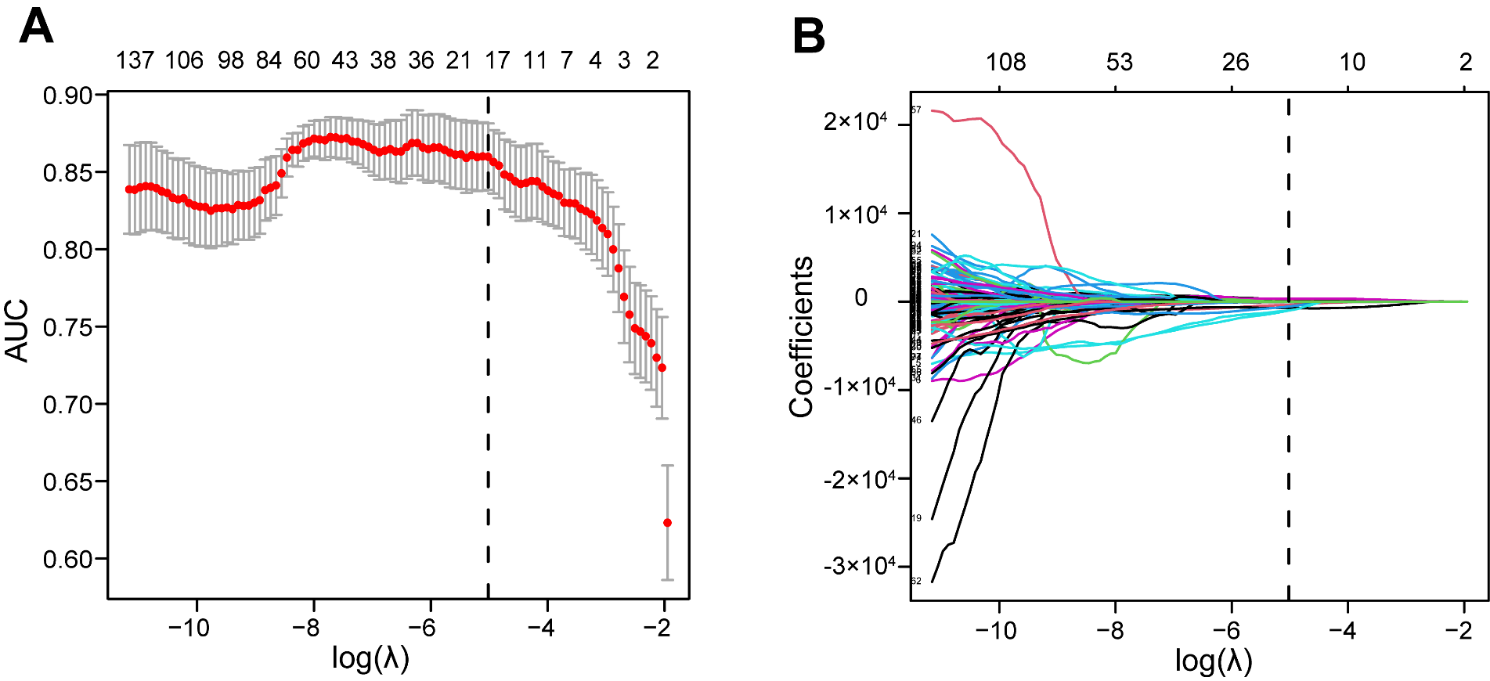


### **Supplementary Figure 4. Collagen feature selection using the LASSO regression model.**

**(A)** Tuning parameter (λ) selection in the LASSO model used 10-fold cross-validation via minimum criteria. The AUC was plotted versus log (λ). Dotted vertical lines were drawn at the optimal values by using the 1-standard error of the minimum criteria (the 1-SE criteria). A λ value of 0.006618952, with log (λ) of -5.017818, was chosen (1-SE criteria) according to 10-fold cross-validation. **(B)** LASSO coefficient profiles of the 284 collagen features. A coefficient profile plot was produced against the log (λ) sequence. A vertical line was drawn at the value selected using 10-fold cross-validation, where optimal λ resulted in sixteen nonzero coefficients. *Abbreviations*: LASSO, least absolute shrinkage and selection operator; AUC, area under the curve; SE, standard error.


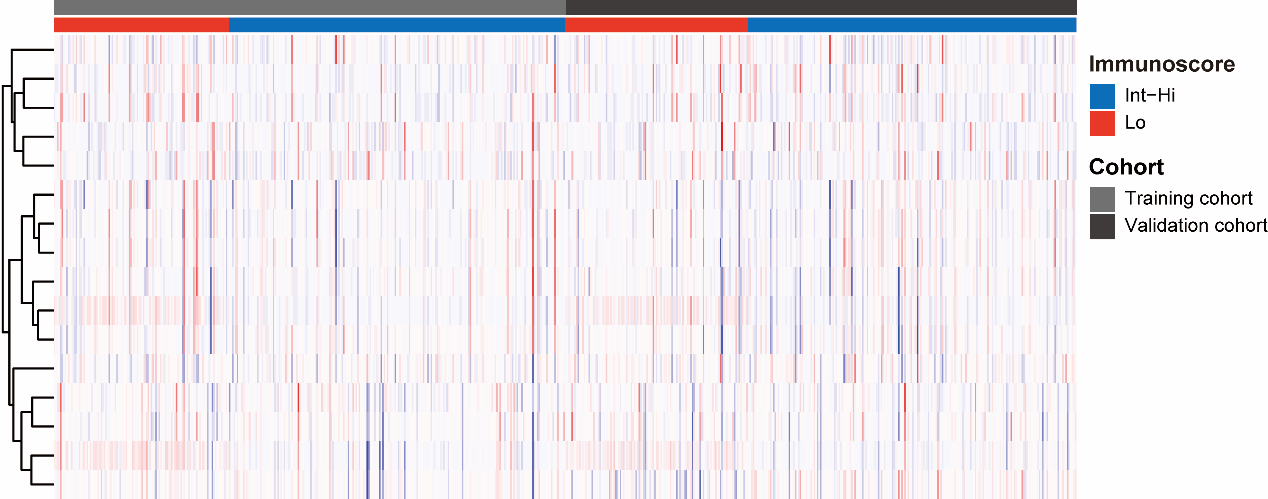


### **Supplementary Figure 5. Collagen features for developing the collagen signature.**

Heatmap of 16 selected collagen features. Each row corresponds to 1 collagen feature, and each column corresponds to 1 patient (separately grouped for the training vs. validation cohort and the low [Lo] vs. intermediate-high [Int-Hi] Immunoscore).


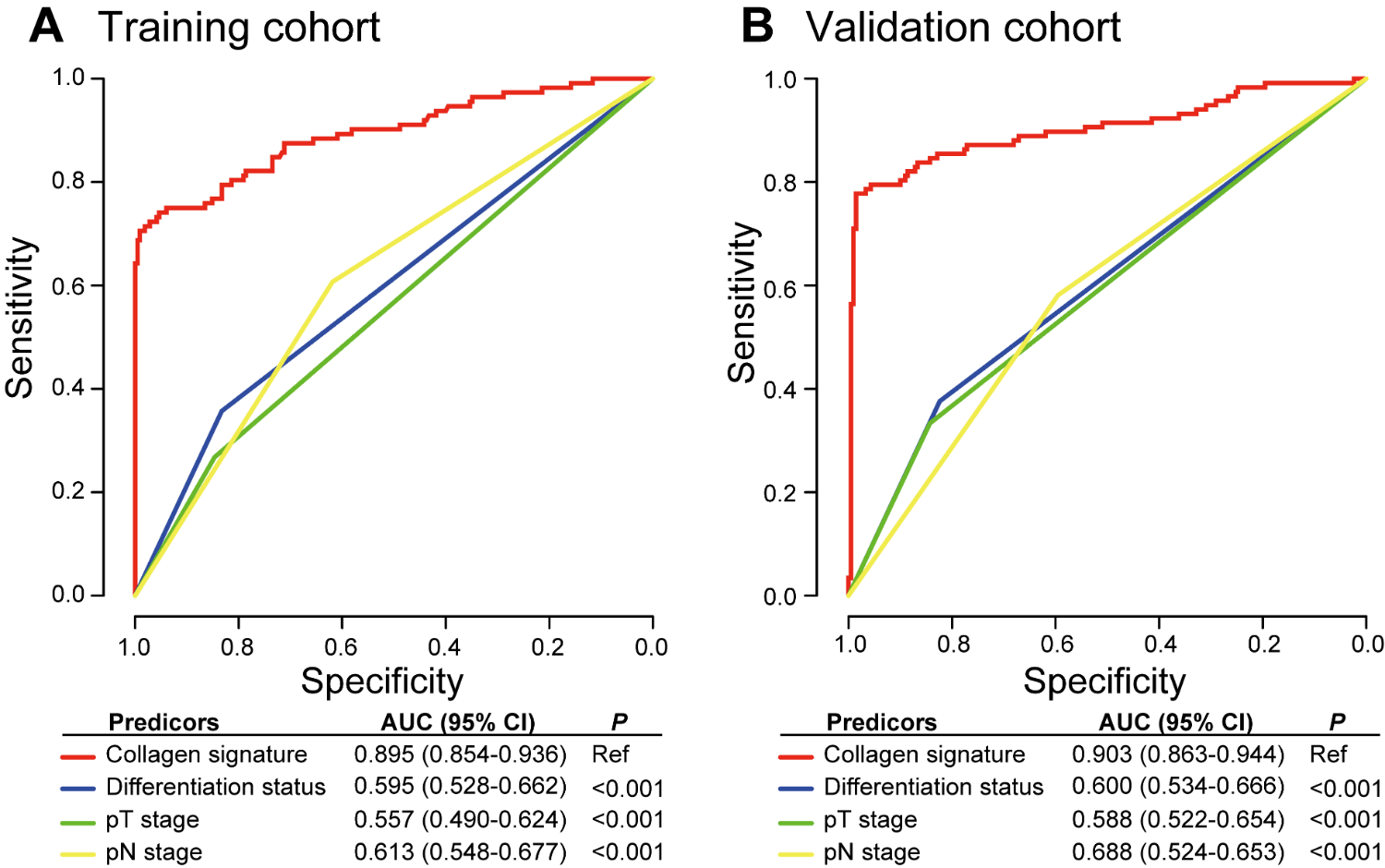


### **Supplementary Figure 6. ROC curves of the predictors for predicting Immunoscore in the training and validation cohorts.**

The collagen signature showed better discrimination than other predictors in the training (A) and validation (B) cohorts. Different variables are represented by different colors. *Abbreviations*: ROC, receiver operating characteristic; AUC, area under the curve; CI, confidence interval.


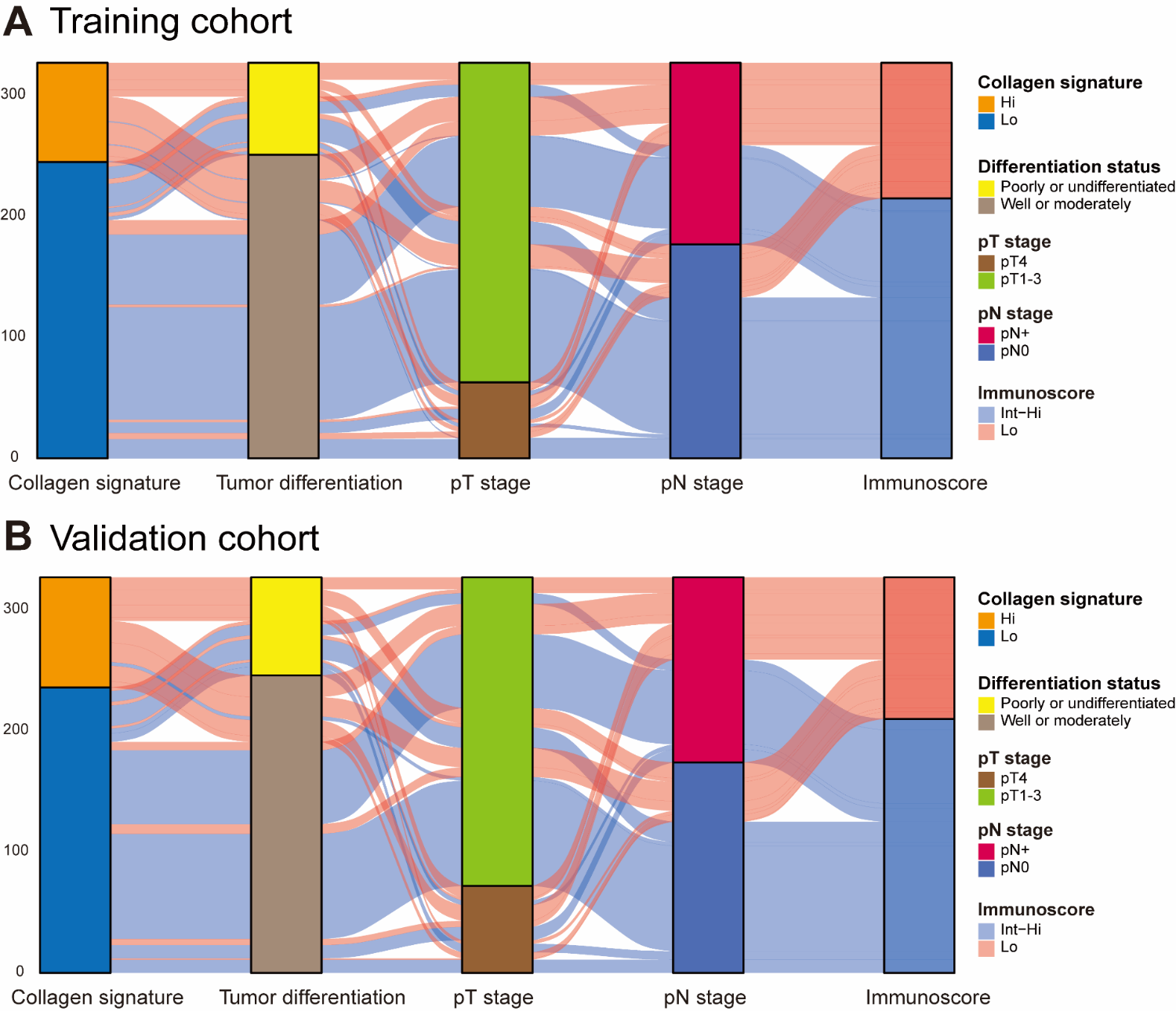


### **Supplementary Figure 7. Alluvial diagrams show the association of predictors with Immunoscore.**

**(A)** Association of four predictors with Immunoscore in the training cohort. **(B)** Association of four predictors with Immunoscore in the validation cohort. *Abbreviations:* Lo, low; Int-Hi, intermediate-high.


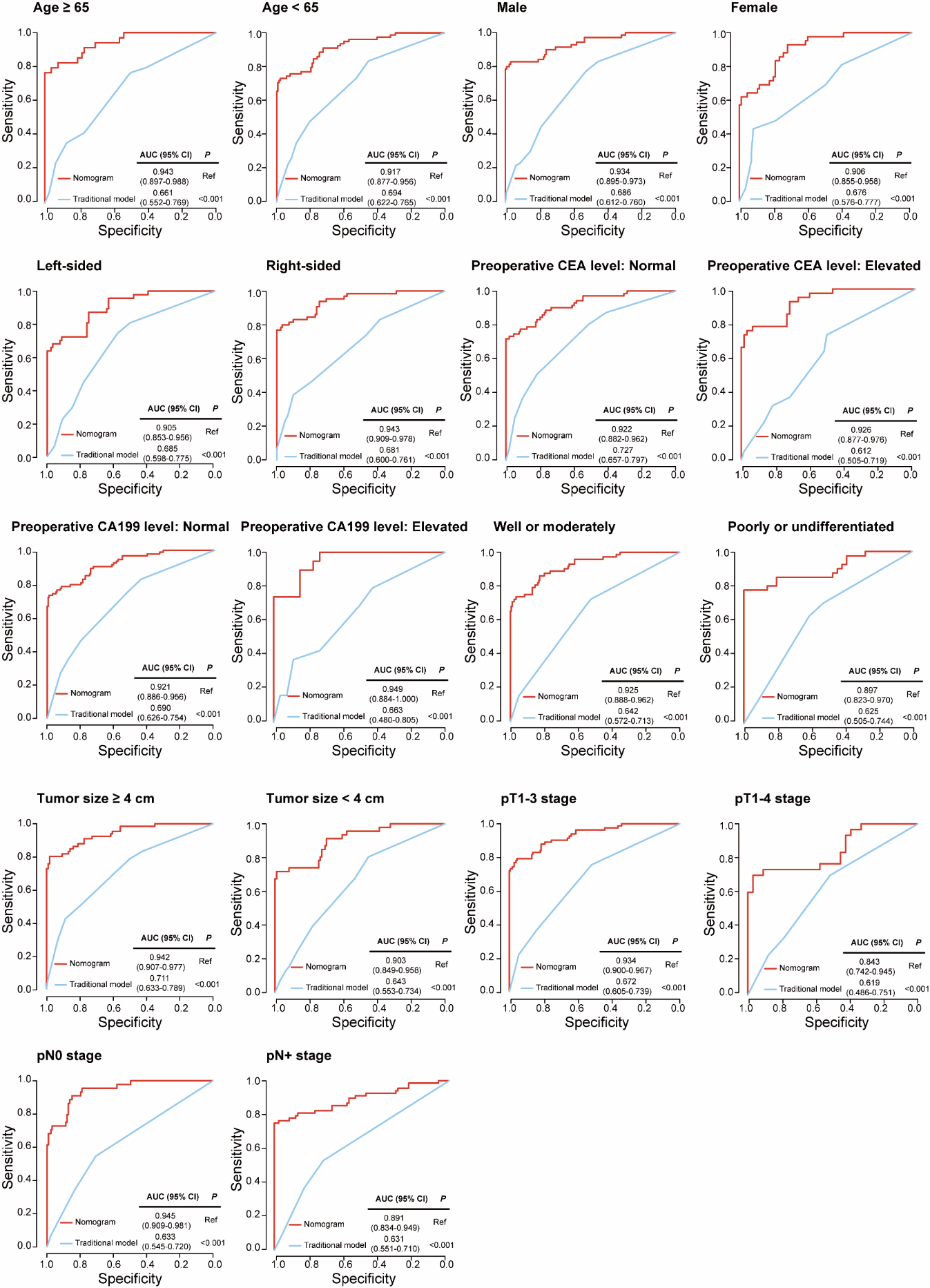


### **Supplementary Figure 8. Stratified analysis of the nomogram and traditional model in the training cohort.**

*Abbreviations:* AUC, area under the curve; CI, confidence interval; Ref, reference.


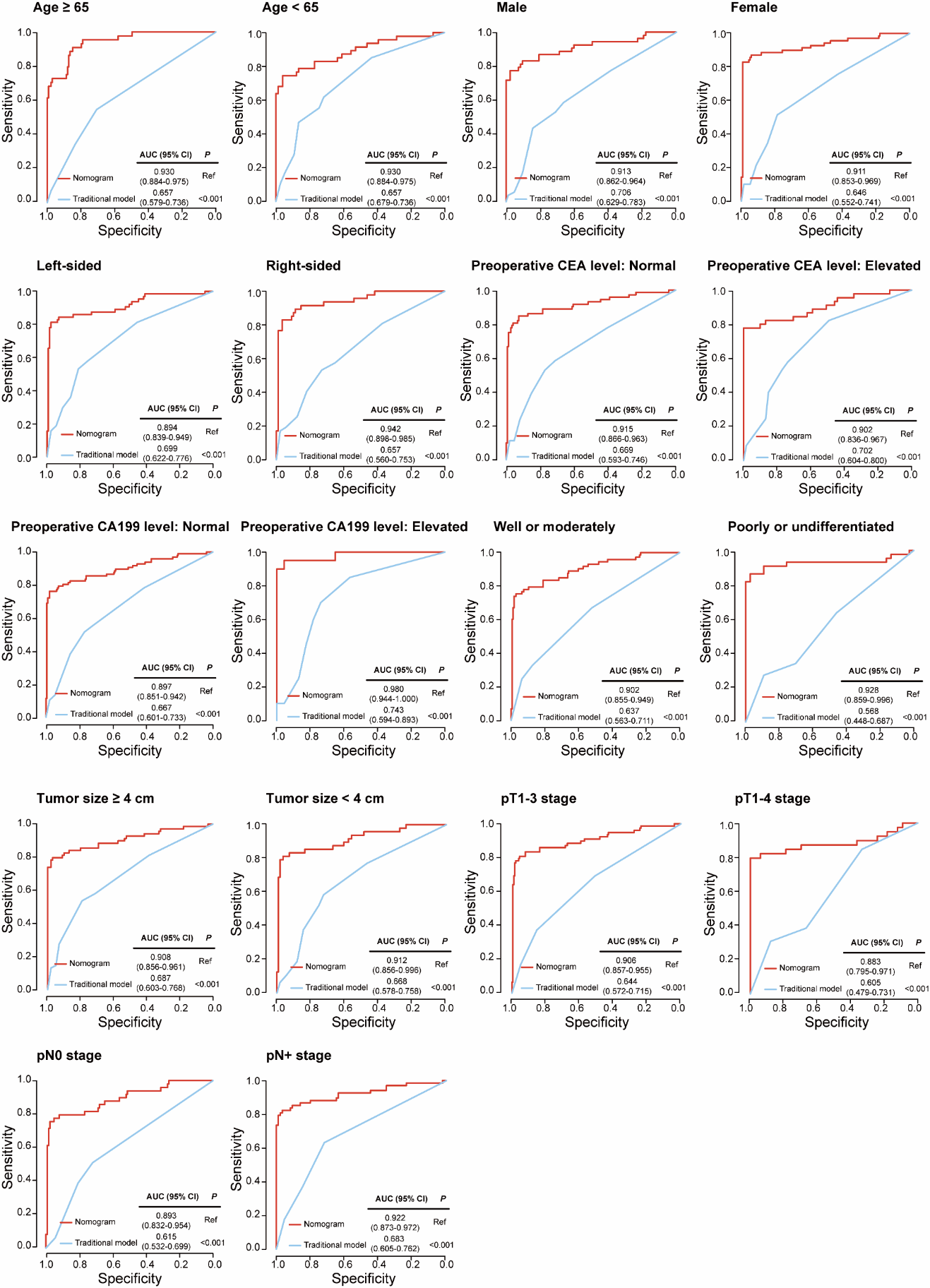


### **Supplementary Figure 9. Stratified analysis of the nomogram and traditional model in the validation cohort.**

*Abbreviations:* AUC, area under the curve; CI, confidence interval; Ref, reference.


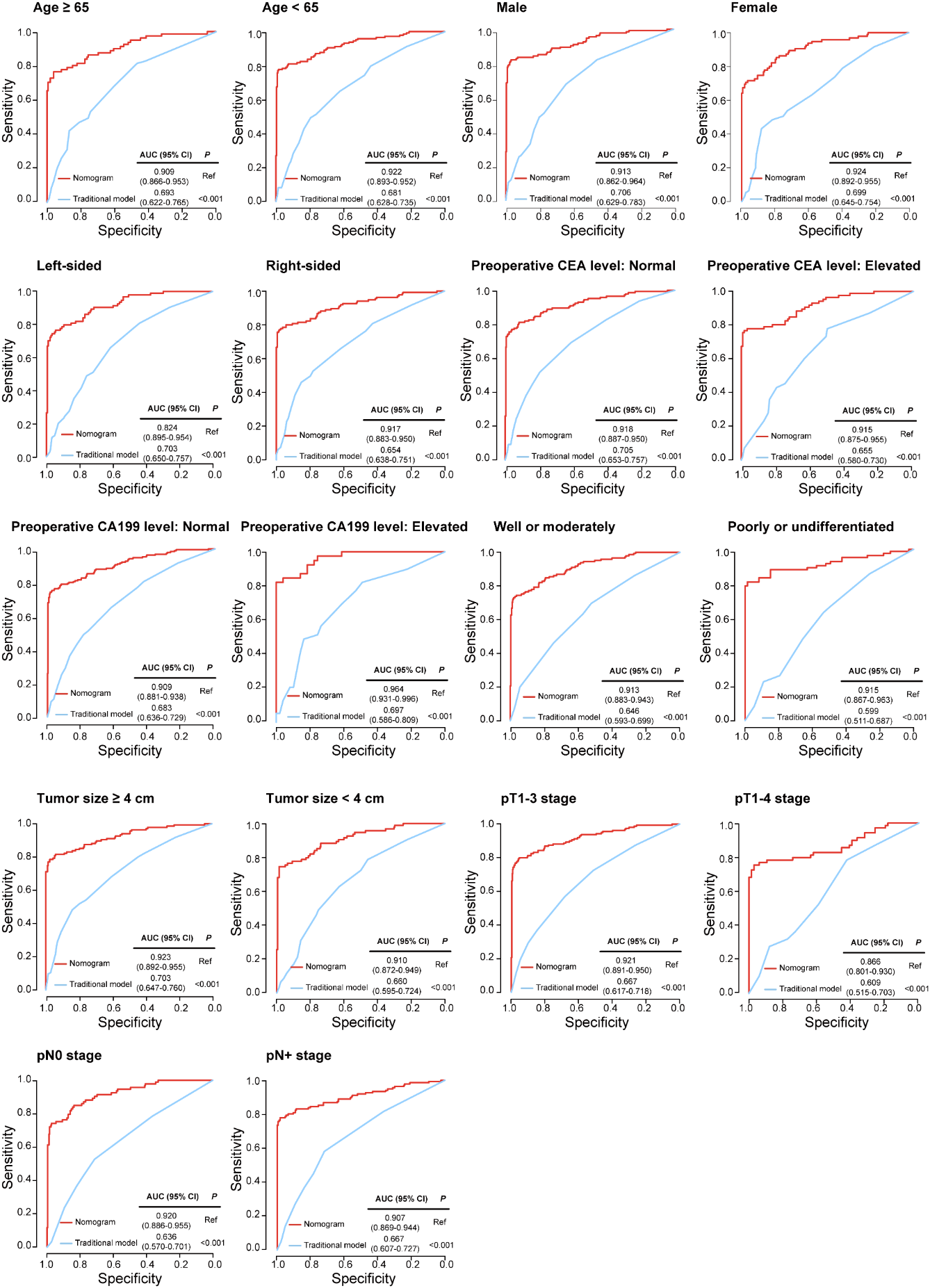


### **Supplementary Figure 10. Stratified analysis of the nomogram and traditional model in all patients.**

*Abbreviations:* AUC, area under the curve; CI, confidence interval; Ref, reference.


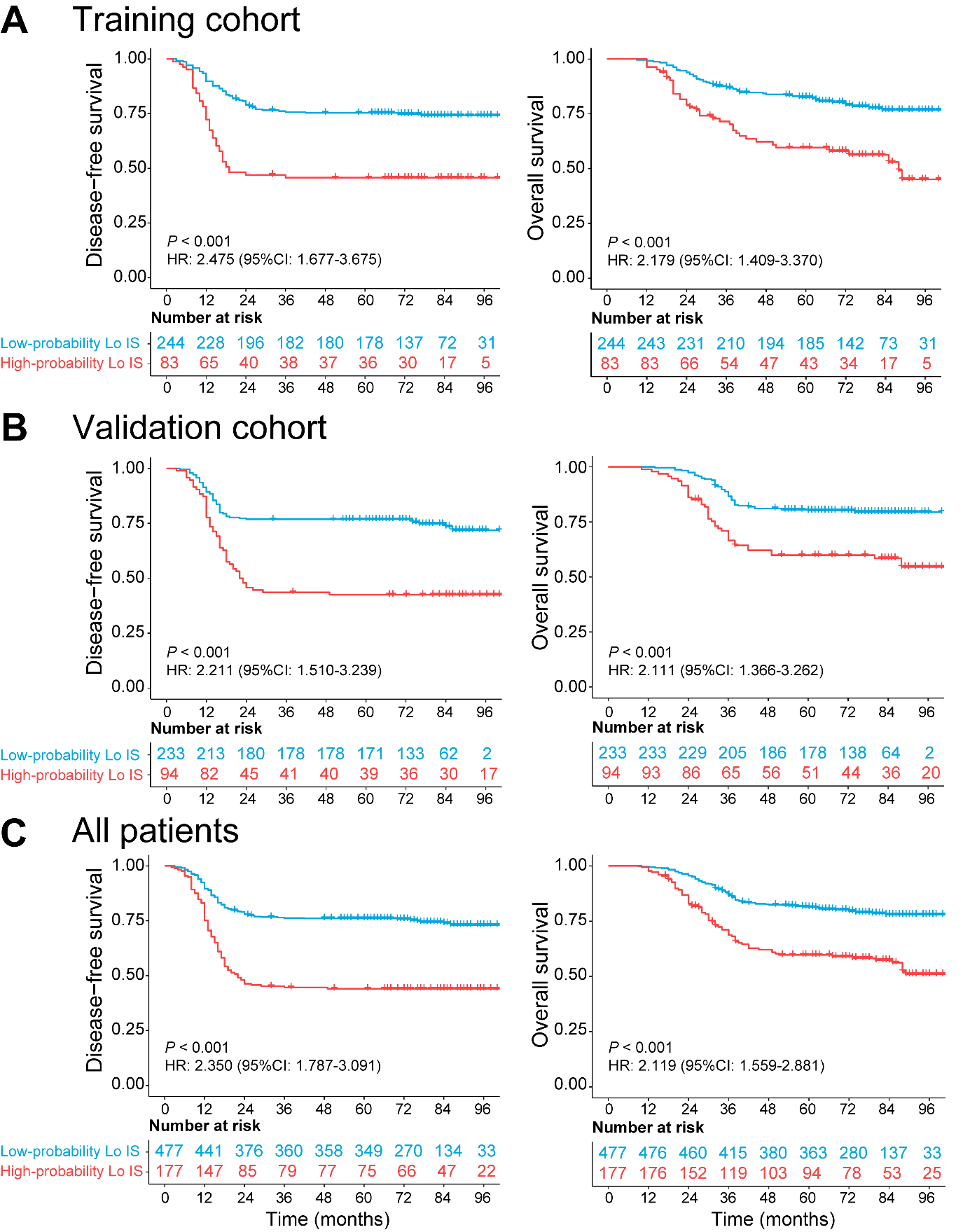


### **Supplementary Figure 11. Kaplan‒Meier analysis of disease-free survival and overall survival in stage I-III patients according to the nomogram-predicted subgroups.**

Disease-free survival and overall survival of the high- and low-probability Lo IS subgroups in the training cohort **(A)**, the validation cohort **(B)**, and all patients **(C)**. *Abbreviations:* Lo IS, low Immunoscore; HR, hazard ratio; CI, confidence interval.


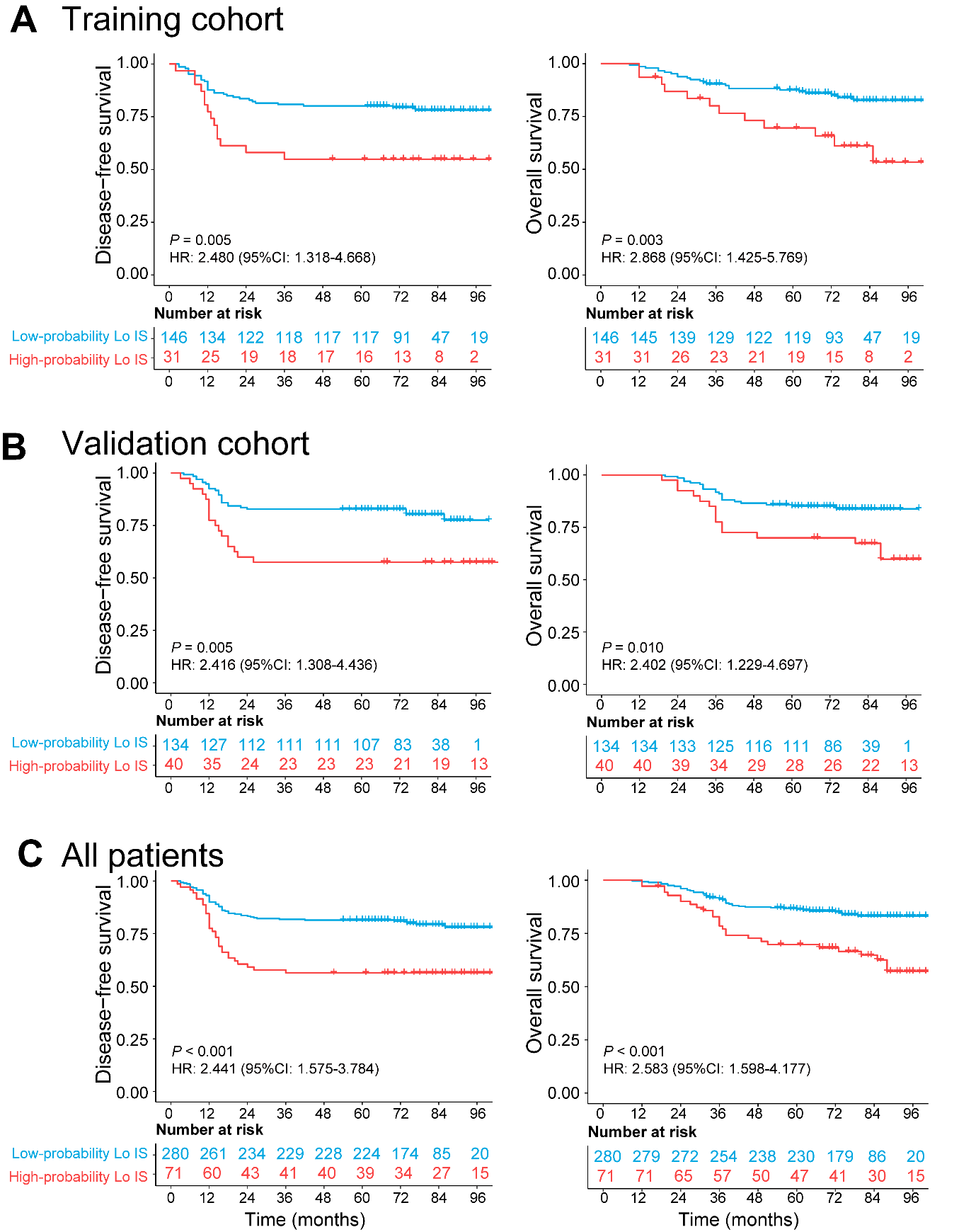


### **Supplementary Figure 12. Kaplan‒Meier analysis of disease-free survival and overall survival in stage I-II patients according to the nomogram-predicted subgroups.**

Disease-free survival and overall survival of the high- and low-probability Lo IS subgroups in the training cohort **(A)**, the validation cohort **(B)**, and all patients **(C)**. *Abbreviations:* Lo IS, low Immunoscore; HR, hazard ratio; CI, confidence interval.


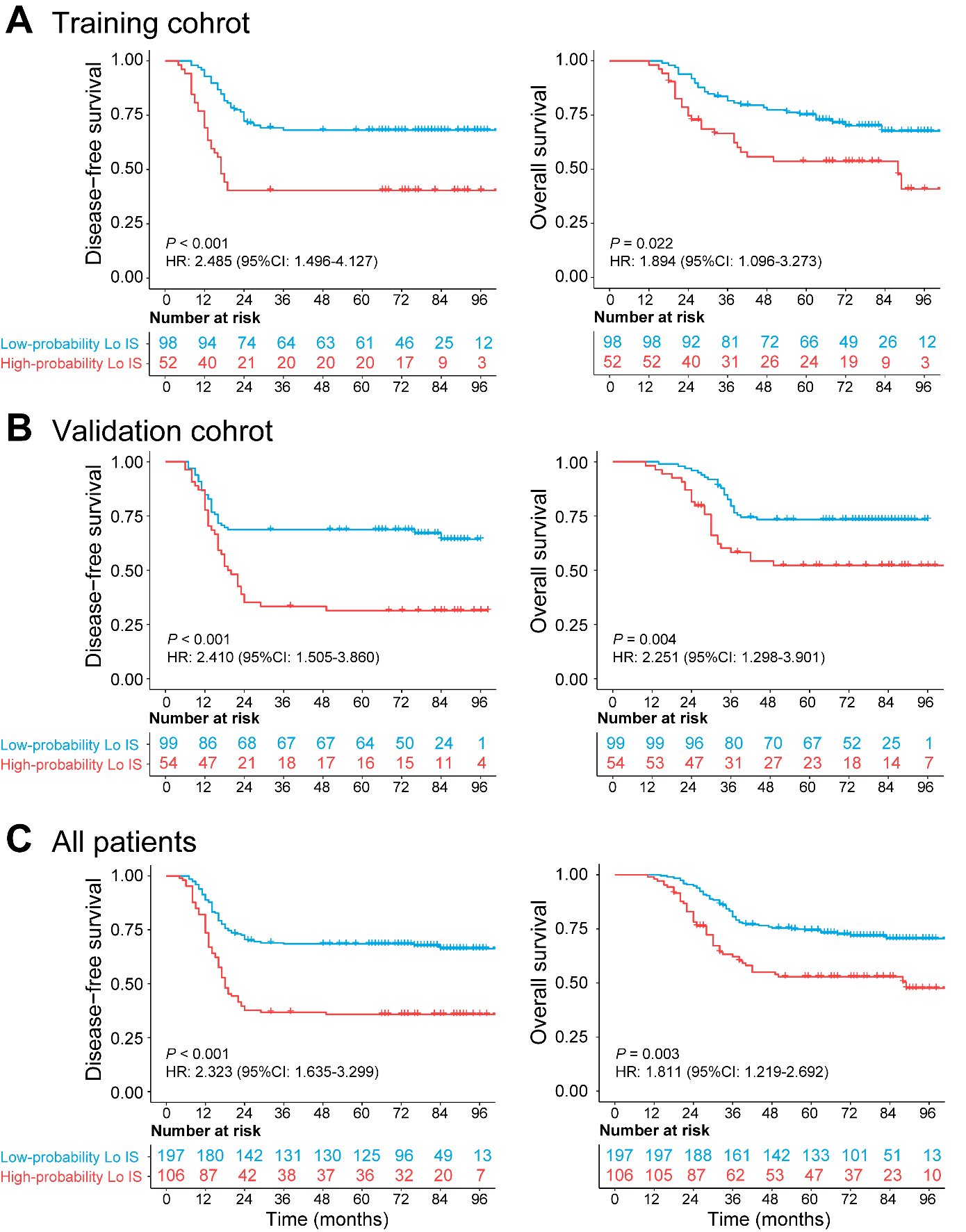


### **Supplementary Figure 13. Kaplan‒Meier analysis of disease-free survival and overall survival in stage III patients according to the nomogram-predicted subgroups.**

Disease-free survival and overall survival of the high- and low-probability Lo IS subgroups in the training cohort **(A)**, the validation cohort **(B)**, and all patients **(C)**. *Abbreviations:* Lo IS, low Immunoscore; HR, hazard ratio; CI, confidence interval.


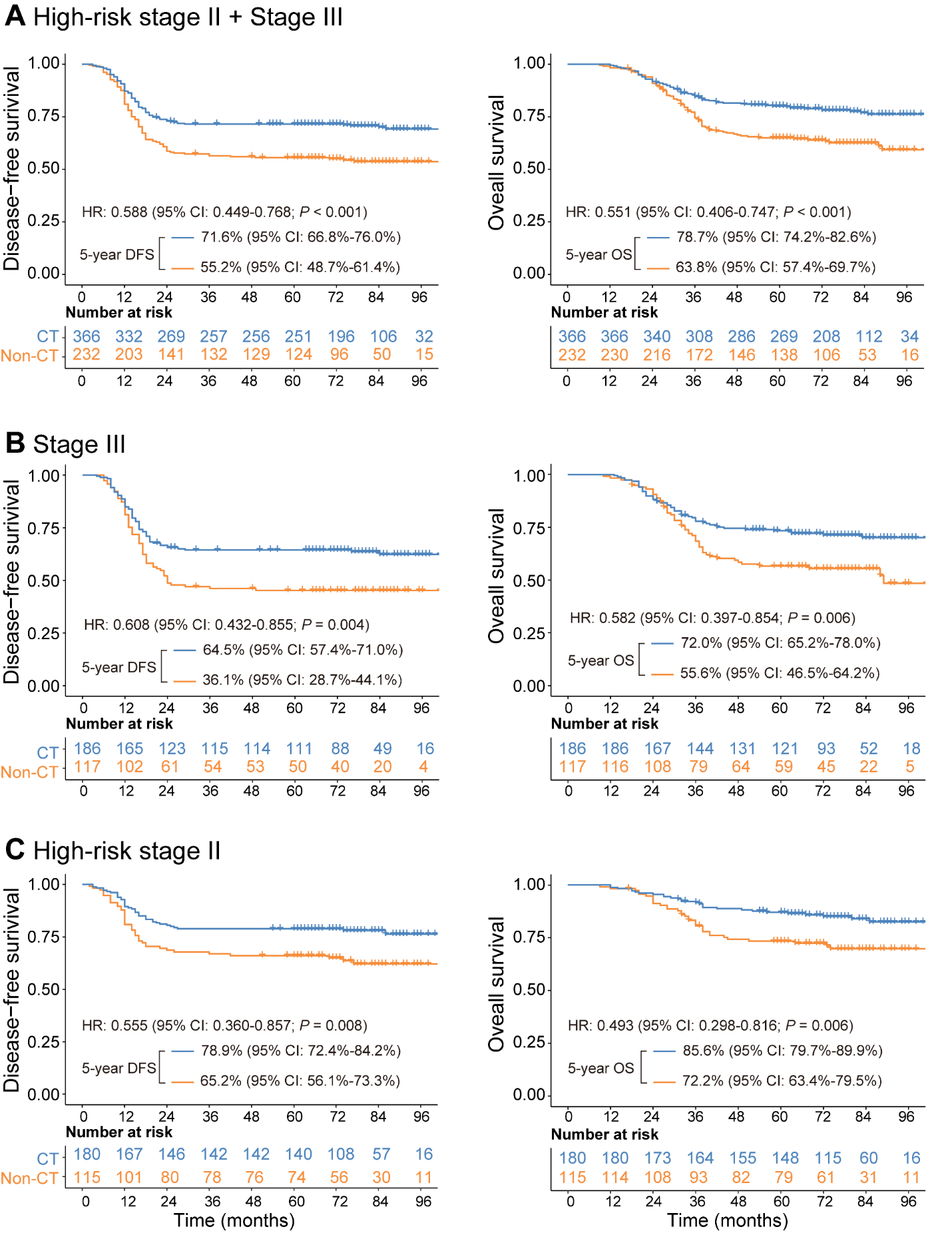


### **Supplementary Figure 14. Kaplan–Meier analyses of DFS and OS according to adjuvant chemotherapy in high-risk stage II and stage III patients.**

*Abbreviations:* CT, chemotherapy; DFS, disease-free survival; OS, overall survival; HR, hazard ratio.

## Supplementary Tables

### **Supplementary Table 1. Extracted collagen features.**

| **No.** | **Collagen feature characterization** |
| --- | --- |
| **Morphological features** | 1.Collagen area |
|  | 2.Collagen number |
|  | 3.Collagen length |
|  | 4.Collagen width |
|  | 5.Collagen straightness |
|  | 6.Collagen crosslink density |
|  | 7.Collagen crosslink space |
|  | 8.Collagen orientation |
| **Texture features** | 9-14.Mean, variance, skewness, kurtosis, energy, entropy of histogram |
|  | 15-95.Contrast, correlation, energy and homogeneity of the Gray-Level Cooccurrence Matrix (GLCM) with four angles and five displacements |
|  | 96-142.Mean and variance of the Gabor filter at four scales with six orientations |

### **Supplementary Table 2. Comparison of clinicopathological characteristics in the training and validation cohorts.**

| **Variables** | **Training cohort**  **(*n* = 327)** | **Validation**  **cohort (*n* = 327)** | ***P*** |
| --- | --- | --- | --- |
| **Age, years** |  |  | 0.462 |
| ≥ 65 | 112 (34.3) | 121 (37.0) |  |
| < 65 | 215 (65.7) | 206 (63.0) |  |
| **Sex** |  |  | 0.524 |
| Male | 199 (60.9) | 191 (58.4) |  |
| Female | 128 (39.1) | 136 (41.6) |  |
| **Primary tumor location** |  |  | 0.751 |
| Left-sided | 187 (57.2) | 191 (58.4) |  |
| Right-sided | 140 (42.8) | 136 (41.6) |  |
| **Preoperative CEA level** |  |  | 0.737 |
| Normal | 225 (68.8) | 221 (67.6) |  |
| Elevated | 102 (31.2) | 106 (32.4) |  |
| **Preoperative CA19-9 level** |  |  | 0.819 |
| Normal | 282 (86.2) | 284 (86.9) |  |
| Elevated | 45 (13.8) | 43 (13.1) |  |
| **Tumor differentiation** |  |  | 0.647 |
| Well or moderately | 251 (76.8) | 246 (75.2) |  |
| Poorly or undifferentiated | 76 (23.2) | 81 (24.8) |  |
| **Tumor size, cm** |  |  | 0.876 |
| < 4 | 155 (47.4) | 157 (48.0) |  |
| ≥ 4 | 172 (52.6) | 170 (52.0) |  |
| **pT stage** |  |  | 0.385 |
| pT1-3 | 264 (80.7) | 255 (78.0) |  |
| pT4 | 63 (19.3) | 72 (22.0) |  |
| **pN stage** |  |  | 0.814 |
| pN0 | 177 (54.1) | 174 (53.2) |  |
| pN+ | 150 (45.9) | 153 (46.8) |  |

Values in parentheses are percentages unless indicated otherwise.

*Abbreviations*: CEA, carcinoembryonic antigen; CA199, carbohydrate antigen 199.

### **Supplementary Table 3. Density (cells/mm2) cutoff values based on the 75th percentile**

|  | **Tumor center** | | | **Invasive margin** | |
| --- | --- | --- | --- | --- | --- |
|  | **CD3 cells/mm^2^** | | **CD8 cells/mm^2^** | **CD3 cells/mm^2^** | **CD8 cells/mm^2^** |
| **Cutoff value (75th percentile)** | 593 | 382 | | 1382 | 714 |
| **Percentiles** |  |  | |  |  |
| **25th** | 204 | 161 | | 554 | 337 |
| **50th** | 400 | 271 | | 863 | 502 |
| **75th** | 593 | 382 | | 1382 | 714 |

### Supplementary Table 4. Stratified analysis of the association between the collagen signature and Immunoscore in the training cohort.

| **Variables** | **Collagen signature, median (IQR)** | | **AUC (95% CI)** | ***P*** |
| --- | --- | --- | --- | --- |
|  | **Lo IS** | **Int-Hi IS** |  |  |
| **Age, years** |  |  |  |  |
| ≥ 65 | 3.094 (0.018, 3.120) | -1.059 (-1.312, -0.863) | 0.927 (0.873, 0.980) | <0.001 |
| < 65 | 2.969 (-0.583, 3.531) | -0.971 (-1.188, -0.799) | 0.879 (0.824, 0.934) | <0.001 |
| **Sex** |  |  |  |  |
| Male | 3.039 (0.644, 3.473) | -0.973 (-1.193, -0.799) | 0.910 (0.858, 0.961) | <0.001 |
| Female | 2.818 (-0.735, 3.519) | -1.012 (-1.296, -0.810) | 0.870 (0.801, 0.938) | <0.001 |
| **Primary tumor location** |  |  |  |  |
| Left-sided | 3.109 (0.326, 3.501) | -1.002 (-1.214, -0.786) | 0.908 (0.857, 0.959) | <0.001 |
| Right-sided | 2.827 (-0.636, 3.432) | -0.973 (-1.234, -0.843) | 0.879 (0.812, 0.946) | <0.001 |
| **Preoperative CEA level** |  |  |  |  |
| Normal | 2.925 (-0.566, 3.485) | -1.009 (-1.226, -0.816) | 0.896 (0.845, 0.946) | <0.001 |
| Elevated | 3.089 (-0.449, 3.456) | -0.939 (-1.222, -0.749) | 0.895 (0.825, 0.965) | <0.001 |
| **Preoperative CA19-9 level** |  |  |  |  |
| Normal | 3.089 (-0.419, 3.504) | -0.978 (-1.210, -0.799) | 0.892 (0.846, 0.938) | <0.001 |
| Elevated | 2.104 (-0.600, 3.316) | -1.102 (-1.412, -0.835) | 0.911 (0.825, 0.997) | <0.001 |
| **Tumor differentiation** |  |  |  |  |
| Well or moderately | 3.078 (-0.618, 3.510) | -0.988 (-1.235, -0.799) | 0.893 (0.844, 0.942) | <0.001 |
| Poorly or undifferentiated | 2.856 (0.275, 3.383) | -0.980 (-1.207, -0.822) | 0.894 (0.819, 0.970) | <0.001 |
| **Tumor size, cm** |  |  |  |  |
| < 4 | 3.078 (-0.575, 3.582) | -0.975 (-1.209, -0.618) | 0.903 (0.850, 0.956) | <0.001 |
| ≥ 4 | 2.924 (-0.361, 3.439) | -0.998 (-1.238, -0.874) | 0.887 (0.827, 0.947) | <0.001 |
| **pT stage** |  |  |  |  |
| T1-3 | 3.018 (0.212, 3.430) | -0.983 (-1.208, -0.810) | 0.903 (0.857, 0.949) | <0.001 |
| T4 | 2.671 (-0.635, 3.582) | -1.061 (-1.454, -0.637) | 0.868 (0.778, 0.958) | <0.001 |
| **pN stage** |  |  |  |  |
| N- | 2.905 (-0.626, 3.256) | -1.008 (-1.256, -0.762) | 0.890 (0.826, 0.954) | <0.001 |
| N+ | 3.197 (-0.160, 3.598) | -0.982 (-1.155, -0.854) | 0.896 (0.841, 0.952) | <0.001 |

*Abbreviations*: Lo, low; Int-Hi, intermediate-high; IS, Immunoscore; IQR, interquartile range; CI, confidence interval; AUC, area under the curve; CEA, carcinoembryonic antigen; CA199, carbohydrate antigen 199.

### **Supplementary Table 5. Stratified analysis of the association between the collagen signature and Immunoscore in the validation cohort.**

| **Variables** | **Collagen signature, median (IQR)** | | **AUC (95% CI)** | ***P*** |
| --- | --- | --- | --- | --- |
|  | **Lo IS** | **Int-Hi IS** |  |  |
| **Age, years** |  |  |  |  |
| ≥ 65 | 2.835 (-0.476, 3.482) | -1.028 (-1.594, -0.868) | 0.878 (0.806, 0.949) | <0.001 |
| < 65 | 3.191 (1.644, 3.663) | -1.038 (-1.385, -0.728) | 0.922 (0.875, 0.970) | <0.001 |
| **Sex** |  |  |  |  |
| Male | 3.143 (0.981, 3.622) | -1.019 (-1.471, -0.736) | 0.911 (0.859, 0.963) | <0.001 |
| Female | 2.835 (0.568, 3.518) | -1.060 (-1.391, -0.828) | 0.897 (0.832, 0.962) | <0.001 |
| **Primary tumor location** |  |  |  |  |
| Left-sided | 3.055 (0.248, 3.566) | -1.029 (-1.584, -0.821) | 0.882 (0.823, 0.942) | <0.001 |
| Right-sided | 3.086 (1.533, 3.601) | -1.038 (-1.276, -0.725) | 0.939 (0.896, 0.983) | <0.001 |
| **Preoperative CEA level** |  |  |  |  |
| Normal | 3.015 (0.795, 3.539) | -1.060 (-1.558, -0.817) | 0.905 (0.854, 0.956) | <0.001 |
| Elevated | 3.103 (0.867, 3.637) | -1.018 (-1.184, -0.727) | 0.896 (0.824, 0.968) | <0.001 |
| **Preoperative CA19-9 level** |  |  |  |  |
| Normal | 3.008 (0.540, 3.532) | -1.045 (-1.470, -0.740) | 0.894 (0.848, 0.940) | <0.001 |
| Elevated | 3.257 (1.583, 3.804) | -1.015 (-1.322, -0.828) | 0.952 (0.872, 1.000) | <0.001 |
| **Tumor differentiation** |  |  |  |  |
| Well or moderately | 2.667 (0.336, 3.551) | -1.029 (-1.471, -0.794) | 0.890 (0.837, 0.942) | <0.001 |
| Poorly or undifferentiated | 3.237 (2.404, 3.658) | -1.072 (-1.239, -0.767) | 0.931 (0.865, 0.996) | <0.001 |
| **Tumor size, cm** |  |  |  |  |
| < 4 | 3.263 (0.864, 3.669) | -1.084 (-1.563, -0.865) | 0.911 (0.853, 0.968) | <0.001 |
| ≥ 4 | 3.000 (0.976, 3.482) | -1.006 (-1.238, -0.629) | 0.897 (0.839, 0.954) | <0.001 |
| **pT stage** |  |  |  |  |
| pT1-3 | 3.004 (0.576, 3.549) | -1.072 (-1.563, -0.840) | 0.912 (0.866, 0.959) | <0.001 |
| pT4 | 3.102 (1.533, 3.678) | -0.817 (-1.051, -0.494) | 0.864 (0.769, 0.959) | <0.001 |
| **pN stage** |  |  |  |  |
| pN0 | 2.815 (1.000, 3.665) | -1.019 (-1.518, -0.817) | 0.887 (0.822, 0.952) | <0.001 |
| pN+ | 3.113 (0.864, 3.539) | -1.089 (-1.389, -0.713) | 0.917 (0.865, 0.969) | <0.001 |

*Abbreviations*: Lo, low; Int-Hi, intermediate-high; IS, Immunoscore; IQR, interquartile range; CI, confidence interval; AUC, area under the curve; CEA, carcinoembryonic antigen; CA199, carbohydrate antigen 199.

### **Supplementary Table 6. Multicollinearity assessment of variables.**

| **Variables** | **Collinearity statistics** | |
| --- | --- | --- |
|  | **Tolerance** | **Variance Inflation Factor** |
| pT stage | 0.992 | 1.008 |
| pN stage | 0.967 | 1.035 |
| Tumor differentiation | 0.976 | 1.025 |
| Collagen signature | 0.944 | 1.059 |

### **Supplementary Table 7. Univariate and multivariable analyses of the predictors of Lo IS without collagen signature in the training cohort.**

| **Variables** | **Univariate analysis** | | **Multivariable analysis** | |
| --- | --- | --- | --- | --- |
|  | **OR (95% CI)** | ***P*** | **OR (95% CI)** | ***P*** |
| **Age, years** |  |  |  |  |
| ≥ 65 | Ref |  |  |  |
| < 65 | 1.306 (0.801, 2.131) | 0.285 |  |  |
| **Sex** |  |  |  |  |
| Male | Ref |  |  |  |
| Female | 0.900 (0.563, 1.440) | 0.660 |  |  |
| **Primary tumor location** |  |  |  |  |
| Left-sided | Ref |  |  |  |
| Right-sided | 0.949 (0.597, 1.506) | 0.823 |  |  |
| **Preoperative CEA level** |  |  |  |  |
| Normal | Ref |  |  |  |
| Elevated | 1.458 (0.897, 2.369) | 0.128 |  |  |
| **Preoperative CA19-9 level** |  |  |  |  |
| Normal | Ref |  |  |  |
| Elevated | 1.485 (0.782, 2.821) | 0.227 |  |  |
| **Tumor differentiation** |  |  |  |  |
| Well or moderately | Ref |  |  |  |
| Poorly or undifferentiated | 2.762 (1.631, 4.678) | <0.001 | 2.693 (1.560, 4.648) | <0.001 |
| **Tumor size, cm** |  |  |  |  |
| < 4 | Ref |  |  |  |
| ≥ 4 | 1.475 (0.930, 2.341) | 0.099 | NA | NA |
| **pT stage** |  |  |  |  |
| pT1-3 | Ref |  |  |  |
| pT4 | 2.018 (1.154, 3.529) | 0.014 | 1.979 (1.098, 3.565) | 0.023 |
| **pN stage** |  |  |  |  |
| pN0 | Ref |  |  |  |
| pN+ | 2.507 (1.569, 4.005) | <0.001 | 2.464 (1.520, 3.994) | <0.001 |

*Abbreviations*: Lo IS, low Immunoscore; OR, odds ratio; CI, confidence interval; CEA, carcinoembryonic antigen; CA199, carbohydrate antigen 199; NA, not available; Ref, reference.

### **Supplementary Table 8. Cox regression analysis of clinicopathological characteristics for survival in the training cohort.**

| **Variables** | **Univariate analysis** | ***P*** | **Multivariable analysis** | ***P*** |
| --- | --- | --- | --- | --- |
|  | **HR (95%CI)** |  | **HR (95%CI)** |  |
| ***Disease-free survival*** | | | | |
| Age (years old) (≥65 vs. <65) | 1.424 (0.934, 2.171) | 0.101 |  |  |
| Sex (Male vs. Female) | 1.108 (0.753, 1.631) | 0.602 |  |  |
| Primary tumor location (Left vs. Right) | 0.828 (0.562, 1.221) | 0.342 |  |  |
| Pretreatment CEA (Elevated vs. Normal) | 1.069 (0.712, 1.606) | 0.747 |  |  |
| Pretreatment CA199 (Elevated vs. Normal) | 1.092 (0.623, 1.915) | 0.759 |  |  |
| Tumor differentiation (Poorly or undifferentiated vs. Well or moderately) | 1.197 (0.777, 1.842) | 0.415 |  |  |
| Tumor size (cm) (≥ 4 vs. < 4) | 1.177 (0.803, 1.724) | 0.404 |  |  |
| pT stage (pT4 vs. pT1-3) | 1.846 (1.210, 2.815) | 0.004 | 1.673 (1.094, 2.560) | 0.018 |
| pN stage (pN+ vs. pN0) | 1.757 (1.197, 2.581) | 0.004 | 1.514 (1.023, 2.241) | 0.038 |
| Probability of Lo IS (High vs. Low) | 2.800 (1.905, 4.117) | <0.001 | 2.475 (1.667, 3.675) | <0.001 |
| ***Overall survival*** | | | | |
| Age (years old) (≥60 vs. <60) | 1.355 (0.858, 2.140) | 0.193 |  |  |
| Sex (Male vs. Female) | 1.143 (0.750, 1.742) | 0.536 |  |  |
| Primary tumor location, (Left vs. Right) | 0.904 (0.593, 1.379) | 0.640 |  |  |
| Pretreatment CEA (Elevated vs. Normal) | 1.361 (0.885, 2.093) | 0.160 |  |  |
| Pretreatment CA199 (Elevated vs. Normal) | 1.458 (0.732, 2.904) | 0.284 |  |  |
| Tumor differentiation (Poorly or undifferentiated vs. Well or moderately) | 1.144 (0.707, 1.852) | 0.583 |  |  |
| Tumor size (cm) (≥ 4 vs. < 4) | 1.307 (0.859, 1.990) | 0.212 |  |  |
| pT stage (pT4 vs. pT1-3) | 2.089 (1.328, 3.284) | 0.001 | 1.900 (1.203, 3.002) | 0.006 |
| pN stage (pN+ vs. pN0) | 2.041 (1.334, 3.125) | 0.001 | 1.763 (1.140, 2.727) | 0.011 |
| Probability of Lo IS (High vs. Low) | 2.607 (1.709, 3.977) | <0.001 | 2.179 (1.409, 3.370) | <0.001 |

*Abbreviations*: HR, hazard ratio; CI, confidence interval; Ref, reference; CEA, carcinoembryonic antigen; CA199, carbohydrate antigen199; Lo IS, low Immunoscore.

### **Supplementary Table 9. Cox regression analysis of clinicopathological characteristics for survival in the validation cohort.**

| **Variables** | **Univariate analysis** | ***P*** | **Multivariable analysis** | ***P*** |
| --- | --- | --- | --- | --- |
|  | **HR (95%CI)** |  | **HR (95%CI)** |  |
| ***Disease-free survival*** | | | | |
| Age (years old) (≥65 vs. <65) | 1.034 (0.706, 1.514) | 0.864 |  |  |
| Sex (Male vs. Female) | 1.082 (0.746, 1.570) | 0.678 |  |  |
| Primary tumor location, (Left vs. Right) | 1.247 (0.861, 1.805) | 0.243 |  |  |
| Pretreatment CEA (Elevated vs. Normal) | 1.272 (0.869, 1.864) | 0.216 |  |  |
| Pretreatment CA199 (Elevated vs. Normal) | 1.350 (0.816, 2.235) | 0.343 |  |  |
| Tumor differentiation (Poorly or undifferentiated vs. Well or moderately) | 1.580 (1.066, 2.340) | 0.023 | NA | NA |
| Tumor size (cm) (≥ 4 vs. < 4) | 1.231 (0.848, 1.786) | 0.274 |  |  |
| pT stage (pT4 vs. pT1-3) | 2.389 (1.627, 3.509) | <0.001 | 1.822 (1.221, 2.721) | 0.003 |
| pN stage (pN+ vs. pN0) | 2.118 (1.448, 3.098) | <0.001 | 1.696 (1.144, 2.515) | 0.009 |
| Probability of Lo IS (High vs. Low) | 2.654 (1.833, 3.843) | <0.001 | 2.211 (1.510, 3.239) | <0.001 |
| ***Overall survival*** | | | | |
| Age (years old) (≥65 vs. <65) | 0.920 (0.593, 1.426) | 0.709 |  |  |
| Sex (Male vs. Female) | 0.960 (0.625, 1.474) | 0.852 |  |  |
| Primary tumor location, (Left vs. Right) | 1.378 (0.905, 2.099) | 0.135 |  |  |
| Pretreatment CEA (Elevated vs. Normal) | 0.984 (0.625, 1.550) | 0.945 |  |  |
| Pretreatment CA199 (Elevated vs. Normal) | 1.291 (0.729, 2.287) | 0.382 |  |  |
| Tumor differentiation (Poorly or undifferentiated vs. Well or moderately) | 1.706 (1.096, 2.655) | 0.018 | NA | NA |
| Tumor size (cm) (≥ 4 vs. < 4) | 1.131 (0.742, 1.725) | 0.567 |  |  |
| pT stage (pT4 vs. pT1-3) | 2.254 (1.448, 3.510) | <0.001 | 1.655 (1.073, 2.865) | 0.010 |
| pN stage (pN+ vs. pN0) | 1.892 (1.234, 2.900) | 0.003 | 1.655 (1.073, 2.552) | 0.023 |
| Probability of Lo IS (High vs. Low) | 2.477 (1.622, 3.782) | <0.001 | 2.111 (1.366, 3.262) | <0.001 |

*Abbreviations*: HR, hazard ratio; CI, confidence interval; NA, not available; Ref, reference; CEA, carcinoembryonic antigen; CA199, carbohydrate antigen199; Lo IS, low Immunoscore.

### **Supplementary Table 10. Cox regression analysis of clinicopathological characteristics for survival in all patients.**

| **Variables** | **Univariate analysis** | ***P*** | **Multivariable analysis** | ***P*** |
| --- | --- | --- | --- | --- |
|  | **HR (95%CI)** |  | **HR (95%CI)** |  |
| ***Disease-free survival*** | | | | |
| Age (years old) (≥65 vs. <65) | 1.160 (0.875, 1.538) | 0.301 |  |  |
| Sex (Male vs. Female) | 1.099 (0.841, 1.436) | 0.490 |  |  |
| Primary tumor location, (Left vs. Right) | 1.019 (0.780, 1.331) | 0.891 |  |  |
| Pretreatment CEA (Elevated vs. Normal) | 1.172 (0.888, 1.548) | 0.262 |  |  |
| Pretreatment CA199 (Elevated vs. Normal) | 1.120 (0.770, 1.629) | 0.654 |  |  |
| Tumor differentiation (Poorly or undifferentiated vs. Well or moderately) | 1.387 (0.038, 1.853) | 0.027 | NA | NA |
| Tumor size (cm) (≥ 4 vs. < 4) | 1.207 (0.924, 1.575) | 0.167 |  |  |
| pT stage (T4 vs. T1-3) | 2.133 (1.607, 2.831) | <0.001 | 1.767 (1.324, 2.359) | <0.001 |
| pN stage (N+ vs. N-) | 1.927 (1.471, 2.524) | <0.001 | 1.607 (1.219, 2.118) | 0.001 |
| Probability of Lo IS (High vs. Low) | 2.742 (2.101, 3.580) | <0.001 | 2.350 (1.787, 3.091) | <0.001 |
| ***Overall survival*** | | | | |
| Age (years old) (≥65 vs. <65) | 1.214 (0.885, 1.664) | 0.230 |  |  |
| Sex (Male vs. Female) | 1.045 (0.773, 1.411) | 0.776 |  |  |
| Primary tumor location, (Left vs. Right) | 1.116 (0.829, 1.502) | 0.469 |  |  |
| Pretreatment CEA (Elevated vs. Normal) | 1.162 (0.851, 1.587) | 0.346 |  |  |
| Pretreatment CA199 (Elevated vs. Normal) | 0.957 (0.617, 1.483) | 0.843 |  |  |
| Tumor differentiation (Poorly or undifferentiated vs. Well or moderately) | 1.405 (1.016, 1.942) | 0.040 | NA | NA |
| Tumor size (cm) (≥ 4 vs. < 4) | 1.216 (0.903, 1.638) | 0.197 |  |  |
| pT stage (pT4 vs. pT1-3) | 2.135 (1.557, 2.929) | <0.001 | 1.802 (1.305, 2.487) | <0.001 |
| pN stage (pN+ vs. pN0) | 1.962 (1.451, 2.652) | <0.001 | 1.709 (1.258, 2.322) | 0.001 |
| Probability of Lo IS (High vs. Low) | 2.507 (1.860, 3.378) | <0.001 | 2.119 (1.559, 2.881) | <0.001 |

*Abbreviations*: HR, hazard ratio; CI, confidence interval; NA, not available; Ref, reference; CEA, carcinoembryonic antigen; CA199, carbohydrate antigen199; Lo IS, low Immunoscore.

# Supplementary References

1. Wang G, Sun Y, Chen Y, Gao Q, Peng D, Lin H, et al. Rapid identification of human ovarian cancer in second harmonic generation images using radiomics feature analyses and tree-based pipeline optimization tool*. J Biophotonics* (2020) 13(9):e202000050. doi:10.1002/jbio.202000050.

2. Dempster AP. Maximum likelihood from incomplete data via the EM algorithm*. J R Stat Soc* (1977) 39:1-38.

3. Stein AM, Vader DA, Jawerth LM, Weitz DA, Sander LM. An algorithm for extracting the network geometry of three-dimensional collagen gels*. J Microsc* (2010) 232(3):463-475. doi:10.1111/j.1365-2818.2008.02141.x.

4. Frisch KE, Duenwald-Kuehl SE, Kobayashi H, Chamberlain CS, Lakes RS, Vanderby R, Jr. Quantification of collagen organization using fractal dimensions and Fourier transforms*. Acta Histochem* (2012) 114(2):140-4. doi:10.1016/j.acthis.2011.03.010.

5. Haralick RM, Shanmugam K, Dinstein I. Textural Features for Image Classification*. Studies in Media and Communication* (1973) SMC-3(6):610-621.

6. Daugman JG. Complete discrete 2-D Gabor transforms by neural networks for image analysis and compression*. IEEE Transacoustspeech & Signal Process* (1988) 36(7):1169-1179.

7. Jian H, Ma S, Zhang CH. Adaptive LASSO for sparse high-dimensional regression*. Stat Sin* (2008) 18(4):1603-1618. doi:10.1007/s11135-007-9120-4.

8. Meier L, Geer SVD, Bhlmann P, Zrich ETH. The group Lasso for logistic regression*. J R Stat Soc B* (2008) 70(1):53-71. doi:10.2307/20203811.

9. Balachandran V, Gonen M, Smith J, DeMatteo R. Nomograms in oncology: more than meets the eye*. Lancet Oncol* (2015) 16(4):e173-80. doi:10.1016/s1470-2045(14)71116-7.

10. El Sharouni MA, Ahmed T, Varey AHR, Elias SG, Witkamp AJ, Sigurdsson V, et al. Development and Validation of Nomograms to Predict Local, Regional, and Distant Recurrence in Patients With Thin (T1) Melanomas*. J Clin Oncol* (2021) 39(11):1243-1252. doi:10.1200/JCO.20.02446.

11. Fitzgerald M, Saville B, Lewis R. Decision curve analysis*. JAMA* (2015) 313(4):409-410. doi:10.1001/jama.2015.37.

12. Vickers AJ, Elkin EB. Decision Curve Analysis: A Novel Method for Evaluating Prediction Models*. Med Decis Making* (2006) 26(6):565-574. doi:10.1177/0272989X06295361.

13. KF K, MD B, K Z, H J. Assessing the Clinical Impact of Risk Prediction Models With Decision Curves: Guidance for Correct Interpretation and Appropriate Use*. J Clin Oncol* (2016) 34(21):2534-40. doi:10.1200/JCO.2015.65.5654.

14. Bragg F, Trichia E, Aguilar-Ramirez D, Besevic J, Lewington S, Emberson J. Predictive value of circulating NMR metabolic biomarkers for type 2 diabetes risk in the UK Biobank study*. BMC Med* (2022) 20(1):159. doi:10.1186/s12916-022-02354-9.

15. Zhou N, Ji Z, Li F, Qiao B, Lin R, Jiang W, et al. Machine Learning-Based Personalized Risk Prediction Model for Mortality of Patients Undergoing Mitral Valve Surgery: The PRIME Score*. Front Cardiovasc Med* (2022) 9:866257. doi:10.3389/fcvm.2022.866257.
